# Subventricular Zone‐on‐a‐Chip: A Model to Study Neurogenesis Disruption in Neonatal Intraventricular Hemorrhage

**DOI:** 10.1002/advs.202502145

**Published:** 2025-10-24

**Authors:** Laura Nicoleti Zamproni, Begüm Gökçe, Magnus Gram, Coco Holliday, Aylin Sendemir, Marimélia Aparecida Porcionatto, Anna Herland

**Affiliations:** ^1^ Department of Biochemistry Escola Paulista de Medicina Universidade Federal de São Paulo São Paulo 04044‐020 Brazil; ^2^ Division of Nanobiotechnology SciLifeLab, Department of Protein Science KTH Royal Institute of Technology Solna 17165 Sweden; ^3^ AIMES – Center for the Advancement of Integrated Medical and Engineering Sciences at Karolinska Institutet and KTH Royal Institute of Technology Stockholm 17164 Sweden; ^4^ Department of Neuroscience Karolinska Institutet Solna 17164 Stockholm Sweden; ^5^ Pediatrics Department of Clinical Sciences Lund Lund University Lund 22184 Sweden; ^6^ Department of Neonatology Skåne University Hospital Lund 22184 Sweden; ^7^ Department of Bioengineering Graduate School of Natural and Applied Sciences Ege University Izmir 35040; ^8^ Department of Bioengineering Faculty of Engineering Ege University Izmir 35040 Türkiye; ^9^ Department of Biomedical Technologies Graduate School of Natural and Applied Sciences Ege University Izmir 35040 Türkiye; ^10^ Biofilms – Research Center for Biointerfaces Department of Biomedical Science Faculty of Health and Society Malmö University Malmö 20506 Sweden; ^11^ Department of Immunology Harvard Medical School Boston MA 02115 USA

**Keywords:** cerebrospinal fluid, inflammation, interleukin‐1b, intraventricular hemorrhage, neurogenesis, organ‐on‐a‐chip, subventricular zone

## Abstract

Intraventricular hemorrhage (IVH) in preterm infants disrupts neurogenesis in the subventricular zone (SVZ), a key neurogenic niche, yet no effective treatments exist. This work develops a human SVZ‐on‐a‐chip model to investigate the inflammatory response in IVH and its impact on neurogenesis. Using this platform, this work examines the effects of red blood cell lysate (RBCL) and hemorrhagic cerebrospinal fluid (CSF) from preterm infants with IVH on SVZ cells. Transcriptomic analysis reveal activation of inflammatory pathways in fetal astrocytes and brain microvascular endothelial cells exposed to hemoglobin isoforms. Notably, interleukin‐1B (IL1B) is upregulated following RBCL and hemorrhagic CSF exposure. To probe its role, this work applies an IL1 receptor antagonist, which demonstrate that IL1B has a partially protective influence on neurogenesis. These findings highlight the SVZ‐on‐a‐chip as a powerful tool for studying IVH pathology and emphasize the role of inflammation in regulating neurogenesis. IL1B emerges as a potential therapeutic target, offering new avenues for intervention. This study advances the understanding of IVH and lays the groundwork for developing strategies to protect the developing brain.

## Graphical abstract

A human **subventricular zone‐on‐a‐chip** is developed to model intraventricular hemorrhage (IVH) in preterm infants. This platform enables the study of inflammation‐driven neurogenic disruption. Exposure to hemorrhagic cerebrospinal fluid and red blood cell lysate activated inflammatory pathways, with IL1B emerging as a key mediator. This study establishes a powerful tool for investigating IVH pathology and potential therapeutic targets. Created with Biorender.com.

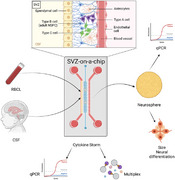

## 1 Background

Intraventricular hemorrhage (IVH), particularly in extremely preterm infants (i.e., born at <28 gestational weeks), presents a significant clinical challenge with lasting neurological consequences.^[^ ^1^, ^2^ ^]^ It affects up to 20% of extremely preterm newborns, and despite advancements in neonatal care, IVH remains a leading cause of morbidity and mortality in neonatal intensive care units.^[^ ^2^ ^]^ ≈50% of extremely preterm survivors of IVH are at risk of developing cerebral palsy or cognitive impairments.^[^ ^3^ ^]^ Currently, there is no specific treatment for IVH beyond intensive care, during which cerebrospinal fluid (CSF) drainage is often used to manage intracranial pressure.^[^ ^4^ ^]^

The subventricular zone (SVZ), a key neurogenic niche along the lateral ventricles, is crucial for postnatal brain development and repair.^[^ ^5^, ^6^ ^]^ It houses neural stem cells (NSCs) that generate new neurons and glial cells, contributing to brain plasticity.^[^ ^6^, ^7^ ^]^ The SVZ's microenvironment, including its interactions with CSF and surrounding vasculature, regulates the balance between NSC quiescence and activation.^[^ ^8^, ^9^ ^]^ However, IVH can disrupt this delicate environment, impairing neurogenesis and leading to adverse developmental outcomes. IVH typically originates from the fragile germinal matrix, a highly vascularized area in the developing brain, and extends into the ventricular system.^[^ ^1^, ^10^ ^]^ The presence of blood in the ventricles triggers an inflammatory response that disrupts the SVZ and hampers neurogenesis.^[^ ^11^, ^12^, ^13^ ^]^ Studies in mice and rabbit models have shown that IVH decreases NSC proliferation and survival, contributing to the neurodevelopmental impairments observed in affected infants.^[^ ^11^, ^13^ ^]^ A detailed understanding of the human‐specific cellular and molecular mechanisms underlying these inflammatory processes and neurogenesis is essential for developing therapies to mitigate the long‐term impact of IVH.^[^ ^11^, ^14^ ^]^

Traditional animal models, including rabbits, dogs, lambs, sheep, pigs, and especially rodents, have provided valuable insights into IVH pathophysiology.^[^ ^12^ ^]^ However, beyond ethical concerns, they often fail to reproduce human‐specific responses due to species differences. Classical in vitro systems, in which single or randomly mixed cell types are cultured in 2D, similarly fall short of capturing the structural and cellular complexity of the SVZ.^[^ ^15^ ^]^ Organotypic forebrain slice cultures from neonatal rabbits have helped model certain aspects of IVH.^[^ ^16^ ^]^ Yet, they lack the human‐specific cellular interactions and dynamic vascular–neural interface critical for SVZ neurogenesis. More recently, organ‐on‐a‐chip technologies have emerged as a powerful alternative, offering physiologically relevant microenvironments that allow controlled investigation of multicellular interactions and compartmentalized processes.^[^ ^17^, ^18^, ^19^ ^]^ Nevertheless, no organ‐on‐a‐chip model has yet recreated the human SVZ under hemorrhagic conditions.

To address this gap, we developed a **human SVZ‐on‐a‐chip** that integrates primary human choroid plexus epithelial cells, fetal astrocytes, brain microvascular endothelial cells, and induced pluripotent stem cell (iPSC)‐derived neural stem cells within a 3D microfluidic system. This platform reconstructs essential features of the neurogenic niche, enabling dynamic interrogation of inflammatory and neurogenic processes. Using red blood cell lysate (RBCL) and cerebrospinal fluid (CSF) from preterm infants with IVH, we demonstrate activation of inflammatory pathways in astrocytes and endothelial cells, particularly in response to hemoglobin isoforms. Leveraging qPCR, transcriptomics, multiplex cytokine analysis, and neurosphere assays, we reveal how IVH impairs neurogenesis in this clinically relevant human model. Collectively, our findings highlight the SVZ‐on‐a‐chip as a first‐of‐its‐kind translational system that not only provides mechanistic insights inaccessible with traditional models but also establishes a platform for identifying therapeutic targets and advancing neonatal neuroprotection strategies.

## 2 Experimental Section

### 2.1 Reagents

DPBS minus Ca^++^ and M^g++^ (14 190 144, Thermo Fischer, Waltham, Massachusetts, USA), DPBS with Ca^++^ and Mg^++^ (14 040 091, Thermo Fischer), Astrocyte Media (AM1801, ScienCell,  Carlsbad, California, USA), TrypLE (12 604 013, Thermo Fischer), Attachment factor protein 1× (S006100, Thermo Fischer), EGM ‐2 MV Microvascular Endothelial Cell Growth Medium‐2 BulletKit (CC‐3202, Lonza, Basel, Switzerland), Endothelial Cell Growth Medium MV2 (C‐22022, PromoCell, Heidelberg, Germany), Epithelial Cell Medium (Innoprot, P60104, Derio, Spain), poly‐L‐lysine (PLL) (Innoprot), DMEM: F12 Glutamax (31331‐028, Thermo Fischer), N2 supplement ( 17502‐048, Thermo Fischer), B27 serum free (11 530 536, Thermo Fischer), FGF (233‐FB, R&D Systems, MN, USA), EGF (E9644, Sigma Aldrich, Burlington, Massachusetts, USA), PLO (P3655, Sigma Aldrich), Laminin (L2020, Sigma Aldrich), Matrigel Growth Factor Reduced (GFR) Basement Membrane Matrix, LDEV‐free (354 230, Corning, Corning, New York, USA), Antibiotic‐Antimycotic (15 240 062, Themo Fischer), idenTx 3 Chip (Aim Biotech, Singapore), Transwells 0.4 µM microporous membrane (833 932 041, Sarstedt, Nümbrecht, Germany), High Pure RNA Isolation Kit (11 828 665 001, Roche, Basel, Switzerland), Clariom S Affymetrix Assay (902 927, Thermo Fischer), High‐capacity RNA‐to‐cDNA kit (4 387 406, Thermo Fischer), TaqMan probes (Applied Biosystems, California, USA), Fast Advanced Master Mix (4 444 557, Applied Biosystems), TRIzol (15 596 026, Thermo Fischer), Chloroform (194 002, MP Biomedicals,  Santa Ana, California, USA), Cell recovery solution (354 253, Corning), Anti‐Adherence Rinsing Solution (0 7010, STEMCELL Technologies, Vancouver, Canada), AggreWell800 24‐well plates (34 811, STEMCELL Technologies), Goat serum (G9023, Sigma Aldrich), Trypan Blue (1 450 021, Bio‐Rad, Hercules, California, USA), LEGENDplex Human Inflammation Panel 1 (13‐plex) assay using a V‐bottom plate (BioLegend, San Diego, California, USA), CellROX Green reagent (C10444,Invitrogen), JC‐1 dye (T3168, Invitrogen) IL1receptor antagonist (SRP3327, Sigma Aldrich).

### 2.2 Blood and Blood Component Preparation

Whole blood (100 mL) was obtained from the Karolinska blood transfusion department (Dnr 2025‐02201‐01). The blood was centrifuged at 2000 g for 15 min to separate the red blood cells (RBCs). The supernatant was discarded, and the RBCs were washed with an equal volume of DPBS (without Ca^++^ and Mg^++^) and centrifuged under the same conditions. This washing procedure was repeated twice. The washed RBCs were then lysed by freezing on dry ice, followed by thawing at room temperature and vortexing at maximum speed for 30 s. The lysing process was repeated three times. The resulting RBCL was centrifuged at 16 000 g for 20 min at room temperature to remove cell membranes. The red blood cell lysate (RBCL) was then aliquoted and stored at −80 °C. For methemoglobin (MetHb) production, RBCL aliquots were incubated in sealed 1.5 mL tubes at 37 °C for 96 h.

### 2.3 Quantification of Oxyhemoglobin and Methemoglobin in RBCL

To quantify oxyhemoglobin (OxyHb) and methemoglobin (MetHb), 100 µL of RBCL was diluted 1:10 in DPBS and analyzed using a spectrophotometer (Infinite 200 PRO, Tecan, Männedorf, Switzerland) at wavelengths of 577 nm and 630 nm (7 to 10 replicates). The absorbance of each blood sample was calculated as the mean of the replicate measurements minus the absorbance of the blank (100 µL DPBS). The concentrations of OxyHb and MetHb in µM were calculated using the Winterbourn.^[^ ^20^ ^]^ formula, adjusted for the dilution factor (10×):

(1) $$-\mathrm{OxyHb}=\left(66*A577\right)-\left(80*A630\right)$$

(2) $$-\mathrm{MetHb}=\left(279*A630\right)-\left(3*A577\right)$$

Two types of RBCL preparations were used in experiments: RBCL enriched in oxyhemoglobin (RBCL/OxyHb) and RBCL enriched in methemoglobin (RBCL/MetHb). The percentages of oxyhemoglobin (OxyHb) and methemoglobin (MetHb) in each preparation are detailed in **Table** **1**. Figure S1A, Supporting Information shows the hemoglobin spectra, and Figure S1B, Supporting Information illustrates the color difference between the two preparations, with the typical red color of OxyHb replaced by the brownish hue of MetHb.

**Table 1:** Percentage of OxyHb and MetHb in the RBCL preparations and the CSF.

	RBCL/OxyHb10% (TW experiment)	RBCL/MetHb 10% (TW experiment)	RBCL/OxyHb 10% (chip experiment)	CSF (chip experiment)
Mean A577	1.97176	0.931818	1.64107	0.5285
Mean A630	0.15717	0.541866	0.02692	0.1414
OxyHb concentration (µM)	117.56	18.15	106.15	23.57
MetHb concentration (µM)	37.93	148.38	2.58	37.86
Total (µM)	155.49	166.53	108.73	61.43

### 2.4 Cell Culture

Human Fetal Astrocytes (HFA) (ScienCell, 1800): HFA were cultured in astrocyte media (AM) supplemented with 2% FBS and 1% astrocyte growth supplement (AGS). Cells were used between passages 6 and 9. When the cells reached confluency, they were passaged at a 1:4 ratio. Briefly, cells were washed with DPBS (without Ca^++^ and Mg^++^) and incubated with TrypLE for 3–4 min. The TrypLE was then neutralized by adding five volumes of cell media, followed by centrifugation at 300 g. The cell pellet was resuspended in complete AM media. No coating was required for culturing HFA.

Primary Human Brain Microvascular Endothelial Cells (HBMEC) (ACBRI 376, Cell Systems, Kirkland, Washington, USA): HBMEC were cultured in EGM‐2 MV Microvascular Endothelial Cell Growth Medium‐2 BulletKit (EGM‐2 MV) or Endothelial Cell Growth Medium MV2 (MV2) in flasks coated with attachment factor. Cells were used between passages 8 and 11. Once cells reached confluency, they were passaged at a 1:4 ratio. Briefly, cells were washed with DPBS (without Ca^++^ and Mg^++^) and incubated with TrypLE for 2 min. The TrypLE was neutralized with five volumes of cell media, centrifuged at 300 g, and resuspended in the appropriate media.

Human Choroid Plexus Epithelial Cells (HCPEpiC) (Science Cell, 1310): HCPEpiC were cultured in Epithelial Cell Medium in flasks coated with poly‐L‐lysine (PLL) at a concentration of 2 µg/cm^2^. Cells were used in passages 5 and 6. Briefly, cells were washed with DPBS (without Ca^++^ and Mg^++^) and incubated with TrypLE for 2 min. The TrypLE was neutralized with five volumes of cell media, centrifuged at 300 g, and the cell pellet was resuspended in the appropriate media.

Human Neuroepithelial Stem Cells (hNSCs): hNSC lines (Control 7, dual‐SMAD neural induction) were obtained from the iPS Core Facility at the Karolinska Institute.^[^ ^21^, ^22^ ^]^ Cells were used between passages 13 and 20. All lines were cultured and passaged in DMEM/F12 Glutamax supplemented with N2 (1:100), B27 (1:1000), 10 ng mL^−1^ bFGF, and 10 ng mL^−1^ EGF (referred to as N2B27 media). The cells were grown in flasks double‐coated with poly‐L‐ornithine (PLO, 20 µg mL^−1^) and laminin (L2020, 1:250). Culture vessels were pre‐treated with PLO overnight, washed twice with DPBS (without Ca^++^ and Mg^++^), and then coated with laminin for an additional overnight incubation. NSCs were passaged at a 1:4 to 1:5 ratio. Briefly, cells were washed with DPBS (without Ca^++^ and Mg^++^) and incubated with TrypLE for 3–4 min. The TrypLE was neutralized by adding five volumes of DMEM/F12 Glutamax, followed by centrifugation at 300 g, and resuspended in N2B27 media. The media was completely replenished every other day.

### 2.5 Culture in Transwells

HBMECs were co‐cultured with HFA in attachment factor‐coated Transwells (TW) (Figure S1C, Supporting Information). HFA were first plated on the underside of the TW membrane (50 000 cells per TW) in 50 µL of AM. The TW inserts were then incubated upside down for 2 h to facilitate astrocyte attachment. After this period, HBMEC (50 000 cells per TW) were seeded on the upper side of the TW membrane, either in inserts containing astrocytes or in inserts without astrocytes. All TW inserts were cultured in EGM‐2 MV media. After 24 h, the media in the lower compartment of the TW inserts was either supplemented with 10% RBCL/OxyHb, 10% RBCL/MetHb, or left untreated. The media was refreshed every 72 h. On day five following RBCL exposure, the TW inserts were washed with DPBS (minus Ca^++^ and Mg^++^). To detach the HBMEC, 100 µL of TrypLE was added to the upper compartment of the TW inserts. The detached HBMECs were collected and centrifuged to recover the cells for RNA extraction. The TW inserts containing HFA on the bottom membrane were then washed with lysis buffer to proceed with RNA extraction from the astrocytes.

### 2.6 RNA Extraction for Transcriptome Assay

For each sample, total RNA was extracted by pooling 4 to 8 TW. RNA extraction was performed using the High Pure RNA Isolation Kit. The quality of the extracted RNA was assessed using a NanoDrop spectrophotometer (Thermo Fisher) and further validated with either a 2100 Bioanalyzer (Agilent Technologies, Santa Clara, California, USA) or a TapeStation RNA ScreenTape (Agilent Technologies). RNA integrity number (RIN) ranged from 9.6 to 10.0.

### 2.7 Transcriptome

The Bioinformatics and Expression Analysis (BEA) core facility at Karolinska Institutet utilized the human Clariom S Affymetrix Assay (Thermo Fisher) to generate whole‐transcriptome expression profiles. The samples analyzed included: Control‐EC, Oxy‐EC, and Met‐EC, which were HBMEC cocultures subjected to different treatments (Control, RBCL/OxyHb, and RBCL/MetHb, respectively); and Control‐HFA, Oxy‐HFA, and Met‐HFA, which were HFA cocultures under the same conditions. For each sample, two transcriptome profiles were produced, except for the Control‐EC sample, which had three profiles generated.

### 2.8 Transcriptome Data Analysis

Differentially expressed genes (DEGs) were identified using the Transcriptome Analysis Console (TAC) software version 4.0 (Thermo Fisher). Raw gene expression data were normalized using the software's standard method (SST‐RMA) before statistical analysis. To account for potential batch effects due to arrays being produced at different times, the analysis was adjusted accordingly. DEGs were determined based on Fold Change (FC) values of less than −2 or greater than 2, with p‐values less than 0.05. For this exploratory analysis, false discovery rates (FDRs) were not considered. FDR values for all genes are provided in the supplementary files. Associations between DEGs were explored using the Search Tool for the Retrieval of Interacting Genes/Proteins (STRING, http://string‐db.org/)^[^ ^23^ ^]^ and Reactome pathways.^[^ ^24^ ^]^ Enrichment analysis was performed separately for downregulated and upregulated genes to identify distinct biological processes and pathways affected by the experimental conditions. The analysis flowchart is provided in Figure S2, Supporting Information.

### 2.9 qPCR Validation of Selected DEG

To validate the main findings from the transcriptome analysis, RNA was extracted from 7 to 9 independent assays using the High Pure RNA Isolation Kit, with each assay representing a pool of 4 to 8 TW. Complementary DNA (cDNA) synthesis was performed using the High‐Capacity RNA‐to‐cDNA Kit on a thermal cycler. TaqMan probes targeting specific genes were incubated with cDNA samples in Fast Advanced Master Mix (Applied Biosystems). The samples were then analyzed using the QuantStudio 5 Real‐Time PCR System (Applied Biosystems). Multiplex quantitative PCR (qPCR) was conducted, and delta threshold cycles (ΔCt) were calculated for each well by subtracting the threshold cycle (Ct) of the target gene from the Ct of GAPDH. The delta delta Ct (ΔΔCt) for each target gene was determined by comparing the ΔCt of experimental samples to that of the control within the same experiment. Fold Change (FC) was computed using the 2^(‐ΔΔCt) method.^[^ ^25^ ^]^ A list of the TaqMan probes used is provided in Table S1, Supporting Information.

### 2.10 Assembling the SVZ‐on‐a‐Chip

The procedure is displayed graphically in Figure S3, Supporting Information. This work employed the idenTx 3 Chip from Aim Biotech (Singapore) for our experiments. Initially, NSCs and HFAs were resuspended in Matrigel at concentrations of 8 million cells mL^−1^ for NSCs and 2.6 million cells mL^−1^ for HFAs. This cell‐Matrigel mixture was used to fill the central channel of the chips, which were then incubated for 10 min to allow the Matrigel to solidify. The lateral channels were filled with a mixture of media termed Media Chip (MC; composed of DMEM/F12 Glutamax, N2 1:100, Antibiotic‐Antimycotic 1:100, B27 1:100) and Matrigel in a 500 µL to 50 mL ratio, and incubated at 37 °C for 30 min. HBMECs were then seeded into the vascular (right) channel at a concentration of 8 million cells mL^−1^, using 15 µL of the cell suspension. The chip was rotated to the left and held in this position for 15 min before being returned to its original orientation. The chip was then incubated overnight with MC plus Matrigel (500 µL to 50 mL) in the neural (left) channel and a 1:1 mixture of MC and MV2 plus Matrigel (500 µL to 50 mL) in the vascular channel. On the following day, a second round of HBMEC seeding was performed, and the chip was rotated to the right position for 15 min and then upside down for 15 min. HCPEpiC were seeded into the neural channel at 2 million cells mL^−1^, and the chip was rotated to the right to facilitate seeding over the central channel. From this point onward, the chips were treated with MC in the neural channel and a 1:1 mixture of MC and MV2 in the vascular channel. On Day 1, the media in the neural channel was replaced with MC (with or without 10% RBCL), while the right channel continued to receive the MC‐MV2 1:1 mixture. Subsequent media changes were performed daily only in the right channel, using a 1:1 mixture of MC‐MV2. The removed media was collected, centrifuged at 16,000 g for 20 min at room temperature to remove cell debris, and 50 µL aliquots were frozen at −20 °C for further analysis. On Day 4, 70 µL of MC was added to the neural channel to compensate for evaporation. On Day 7, the chips were either treated with Trizol for RNA extraction of all cells or used for a neurosphere assay.

### 2.11 SVZ‐on‐a‐Chip Immunocytochemistry

Chips were first washed with DPBS (with Ca^++^ and Mg^++^) and then fixed with 4% paraformaldehyde (PFA) overnight at 4 °C. After four washes with DPBS (with Ca^++^ and Mg^++^), the chips were incubated for 1 h with a blocking buffer consisting of 10% goat serum (Sigma Aldrich) and 0.1% Triton X‐100 in DPBS ((with Ca^++^ and Mg^++^). Primary antibody incubation was carried out in a dilution buffer (10% blocking buffer) overnight at 4 °C. Following four washes with DPBS (with Ca^++^ and Mg^++^), chips were incubated with a secondary antibody and DAPI (4′,6′‐diamidino‐2‐phenylindole) (1:2000) in DPBS (with Ca^++^ and Mg^++^) overnight at 4 °C. After three washes in DPBS (with Ca^++^ and Mg^++^), the chips were imaged using a Cell Observer fluorescent microscope (Zeiss, Oberkochen, Germany) or Zeiss LSM900‐Airy 2 confocal. Images were processed using Zeiss Zen blue Software, Imaris (Oxford Instruments, Oxford, UK) or Image J. A detailed list of the antibodies used is provided in Table S2, Supporting Information.

### 2.12 Neurosphere Assay

To perform the NSF recovery assay, we first washed the chips once with DPBS (minus Ca^++^ and Mg^++^). We then added the Cell recovery solution (to depolymerize Matrigel) and incubated at 4 °C for 10 min. The channels were washed with DPBS (minus Ca^++^ and Mg^++^), and as much of the washing DPBS as possible was collected. The samples were centrifuged at 300 g for 5 min, and the supernatant was aspirated. The pellet was resuspended in N2B27 complete media and passed through a 40 µm cell strainer to remove undigested Matrigel pieces. Cells were counted using Trypan Blue. In an Agrewell 800, previously coated with Anti‐Adherence solution (1 h at room temperature and centrifuged at 2000 g for 5 min), we added 200 000 to 250 000 cells mL^−1^ (500 µL) for both groups and centrifuged at 300 g for 5 min. The Agrewell was then transferred carefully to the incubator. The next day, 500 µL of fresh N2B27 media was added. On Day 2, the spheres were transferred from the Agrewell to a six‐well plate, previously coated with Anti‐Adherence solution (1 h at room temperature), and 1 mL of fresh N2B27 media was added. From this point forward, half of the media was changed daily by collecting 1 mL, centrifuging at 300 g for 5 min, discarding the supernatant, and replacing it with 1 mL of fresh N2B27 media. On Day 6, the neurospheres were seeded in PLO‐L2020‐coated 24‐well plates and incubated for 48 h with N2B27 media, excluding EGF and FGF. After 48 h, the neurospheres were either fixed or lysed for RNA collection.

### 2.13 CSF

Hemorrhagic CSF from preterm infants with IVH (gestational age at birth 25–28 weeks) was sampled after detection of germinal matrix (GM)‐IVH, by spinal tap or ventricular reservoir puncture according to clinical routine in the neonatal unit at Lund University Hospital, Lund, Sweden. Immediately after sampling, the CSF was centrifuged at 2000 g, 20 °C for 10 min, pooled and the proportion of OxyHb and MetHb was determined as described above. Samples were stored at − 80 °C until further use, as described below. The undiluted CSF was added to the neural channels of the chips (in line with that described for RBCL), and the subsequent protocols were carried out as described previously.

### 2.14 qPCR

Total RNA was extracted from the chips using Trizol/chloroform and from neurospheres using the High Pure RNA Isolation Kit (Roche). cDNA synthesis was performed with the High‐Capacity RNA‐to‐cDNA Kit (Thermo Fisher) on a thermal cycler. TaqMan probes of interest (Applied Biosystems, CA, USA) were then incubated with the cDNA samples in Fast Advanced Master Mix (Applied Biosystems). The samples were analyzed on the QuantStudio 5 Real‐Time PCR System. Quantification of target genes was normalized using the geometric mean of Beta‐actin (Actb) and Glyceraldehyde 3‐phosphate dehydrogenase (GAPDH) as reference genes. Threshold cycles (Ct) were determined for each sample, and the relative expression of mRNA was calculated using the 2‐ΔΔCt method.^[^ ^25^ ^]^ The TaqMan probes used are listed in Table S1, Supporting Information.

### 2.15 Neurosphere Diameter Measurement

On Day 6, before attachment, neurospheres were photographed using an optical microscope (CKX41, Olympus, Shinjuku, Japan). The diameter of each neurosphere was measured twice using ImageJ software. The average of these two measurements was considered the final diameter of the neurosphere.

### 2.16 Neurosphere Immunocytochemistry

Neurospheres were first washed with DPBS (with Ca^++^ and Mg^++^) and then fixed with 4% paraformaldehyde (PFA) for 10 min at room temperature. After two washes with DPBS (with Ca^++^ and Mg^++^), the neurospheres were incubated with a blocking buffer consisting of 10% goat serum (Sigma Aldrich) and 0.1% Triton X‐100 in DPBS (with Ca^++^ and Mg^++^). Primary antibody incubation was carried out in a dilution buffer (10% blocking buffer) overnight at 4 °C. Following this, neurospheres were incubated with a secondary antibody and DAPI (4′,6′‐diamidino‐2‐phenylindole) (1:2000) in DPBS (with Ca^++^ and Mg^++^) at room temperature for 1 h. After three washes in DPBS (with Ca^++^ and Mg^++^), the neurospheres were imaged using a Cell Observer fluorescent microscope (Zeiss, Oberkochen, Germany). Images were processed using ImageJ software. A detailed list of the antibodies used is provided in Table S2, Supporting Information.

### 2.17 Analysis of Conditioned Media Collected from the Chip Vascular Channel

To analyze cytokine levels in the conditioned media collected from the vascular channels of the chips, we employed the LEGENDplex Human Inflammation Panel 1 (13‐plex). The panel included 13 inflammatory cytokines: IL‐1β, IFN‐α2, IFN‐γ, TNF‐α, MCP‐1, IL‐6, CXCL8 (IL‐8), IL‐10, IL‐12p70, IL‐17A, IL‐18, IL‐23, and IL‐33. Cytokine levels were assessed from pooled samples of four RBCL chips (paired with four controls) and eight CSF chips (paired with eight controls) at days 0, 1, 3, 5, and 7. The assay plate was analyzed using the BD FACSCanto II Flow Cytometry System (BD, Franklin Lakes, New Jersey, USA), which identified bead types and measured fluorescence intensity. Data were then processed with LEGENDplex software to generate standard curves and quantify analyte concentrations.

### 2.18 Oxidative Stress Detection with CellROX

After 6 days, Neurospheres attached to PLO + laminin‐coated 24‐well plates were incubated with CellROX Green reagent at a final concentration of 5 µM in the appropriate culture medium for 30 min at 37 °C, protected from light. Following incubation, cells were washed twice with DPBS minus Ca^++^ and Mg^++^ and immediately processed for fluorescence microscopy. Images were acquired using a Zeiss Cell Observer fluorescence microscope equipped with filters suitable for CellROX Green detection. Images were processed using ImageJ software.

### 2.19 Mitochondrial Membrane Potential Assessment with JC‐1

After 6 days, Neurospheres attached to PLO + laminin‐coated 24‐well plates were incubated with JC‐1 dye at a final concentration of 10 µM in the appropriate culture medium for 30 min at 37 °C, protected from light. After incubation, cells were washed twice with DPBS minus Ca^++^ and Mg^++^ and immediately analyzed by fluorescence microscopy. Images were captured using a Zeiss Cell Observer fluorescence microscope with filters optimized for detection of the JC‐1 monomeric form (green, ≈530 nm) and aggregated form (red, ≈590 nm), corresponding to mitochondrial membrane depolarization and intact membrane potential, respectively. Images were processed using ImageJ software.

### 2.20 Interlukin‐1 Receptor Antagonist (IL‐1RA) Experiment

SVZ‐on‐a‐chip devices were assembled as previously described. Four experimental groups were established:

1. **Control group**, receiving MC in the neural channel and a 1:1 mixture of MC and MV2 in the vascular channel;
2. **RBCL group**, receiving MC + RBCL in the neural channel and a 1:1 mixture of MC and MV2 in the vascular channel;
3. **IL1RA group**, receiving MC in the neural channel and a 1:1 mixture of MC and MV2 supplemented with IL‐1RA (100 ng mL^−1^) in the vascular channel;
4. **RBCL + IL1RA group**, receiving MC + RBCL in the neural channel and a 1:1 mixture of MC and MV2 with IL‐1RA (100 ng mL^−1^) in the vascular channel.

IL‐RA was freshly replaced daily in the vascular channel, as previously described. After 6 days of incubation, neurospheres (NSF) were photographed for diameter measurements. On day 7, NSFs were recovered and seeded onto PLO/laminin‐coated 24‐well plates for RNA extraction and immunostaining with Doublecortin (DCX) and βIII‐tubulin (TUBB3).

### 2.21 Statistical analysis

Data were analyzed using GraphPad Prism 6.0 (GraphPad Software, USA) and are presented as means ± standard error of the mean (SEM). Statistical analyses were conducted using the Student's *t*‐test for normally distributed data and the Mann‐Whitney test or Wilcoxon Signed‐Rank Test for non‐normally distributed data, as verified by the Shapiro‐Wilk test. Statistical significance was accepted at *p* < 0.05.

#### 2.21.1 Ethics Approval and Consent to Participate

CSF sampling was performed following written consent from the parents, and the study was approved by the ethical committee review board for studies in human subjects at Lund University, Lund, Sweden (Dnr. 2009/402).

## 3 Results

### 3.1 Preliminary Investigation of RBCL Effects on Neurogenic Niche Supporting Cells

Before developing our SVZ‐on‐a‐chip model, we conducted experiments to determine whether different hemoglobin isoforms elicit similar or distinct responses in supporting cells of the neurogenic niche. Based on previous findings suggesting that MetHb may induce a stronger inflammatory response than OxyHb, we used two RBCL preparations, each enriched with one of these isoforms.^[^ ^26^ ^]^ Our goal was to identify key inflammatory mediators affected by RBCL exposure, as these factors could critically influence the behavior of NSCs, including their maintenance, proliferation, and differentiation in a damaged niche environment.

To test this, we employed a transwell co‐culture system to perform transcriptomic analysis on primary HFA and primary HBMEC. While choroid plexus cells are integral to the brain's immune response, we prioritized astrocytes and endothelial cells for their accessibility and relevance to the SVZ niche. This approach allowed us to explore fundamental regulators of the niche's structural and functional integrity while maintaining experimental practicality.

### 3.2 HFA Transcriptome Exploratory Analysis

**Figure 1:**
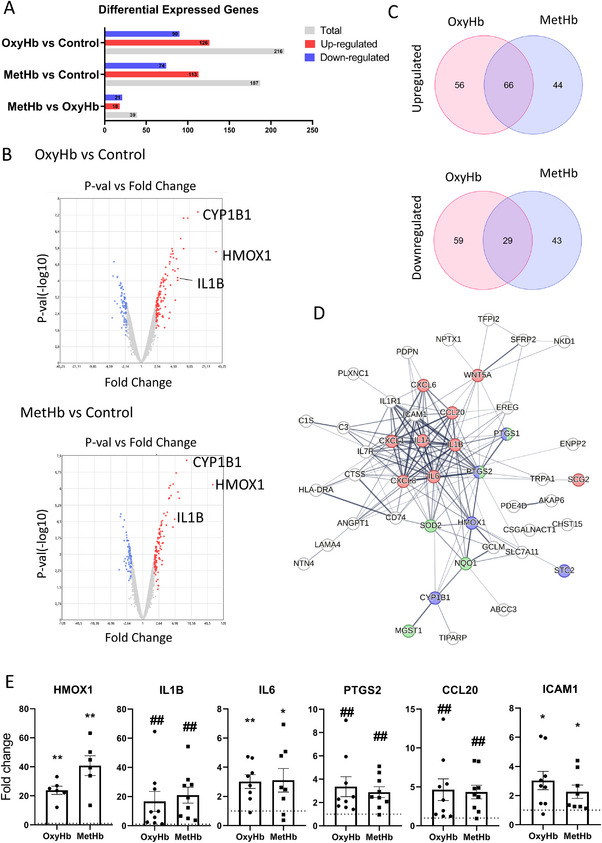
Differential Gene Expression and Functional Interactions in human fetal astrocytes (HFA), OxyHb‐HFA, and MetHb‐HFA Conditions Compared to Control‐HFA. A) Number of differentially expressed genes (DEGs) in the OxyHb‐HFA and MetHb‐HFA groups compared to the Control‐HFA group. B) Volcano plot depicting the distribution of DEGs between the OxyHb‐HFA and MetHb‐HFA groups. Created with Transcriptome Analysis Console (TAC) 4.0, Thermo Fischer. C) Venn diagrams showing the overlap of upregulated and downregulated DEGs unique to OxyHb, unique to MetHb, and common to both conditions. Created with https://molbiotools.com/. D) Protein‐protein interaction network of the upregulated genes in both OxyHb‐HFA and MetHb‐HFA conditions. Nodes are color‐coded by function: blue for heme‐binding, pink for cytokine activity, and green for antioxidant activity. The thickness of the connecting lines represents the strength of data support from text mining, experiments, databases, and co‐expression analyses. Created with STRING (https://string‐db.org/). E) Validation of key genes through qPCR analysis. Results were considered statistically significant when the fold change differed from 1 (control level). *p < 0.05, **p < 0.01 (one‐sample Student's *t*‐test), #*p* < 0.05, ##*p* < 0.01 (Wilcoxon signed‐rank test, used for groups not following a normal distribution as determined by the Shapiro‐Wilk test). No significant statistical difference was observed between the OxyHb and MetHb groups. Results from 9 independent assays. CYP1B1: Cytochrome P450 Family 1 Subfamily B Member 1, HMOX1: Heme Oxygenase 1, IL1B: Interleukin 1 Beta, IL6: Interleukin 6, CCL20: C‐C Motif Chemokine Ligand 20, PTGS2: Prostaglandin‐Endoperoxide Synthase 2, and ICAM1: Intercellular Adhesion Molecule 1.

In the exploratory transcriptome analysis of HFA, co‐cultured with HBMEC and exposed to RBCL, we identified significant differential gene expression (DEG) patterns in both OxyHb‐ and MetHb‐RBCL conditions compared to the control. Specifically, 216 DEGs were identified in the OxyHb‐HFA group (126 upregulated and 90 downregulated) and 187 DEGs in the MetHb‐HFA group (113 upregulated and 74 downregulated) (**Figure** **1**A, File S1, Supporting Information). The distribution of these DEGs is visually represented in the volcano plots shown in Figure 1B.

To better understand the specific and shared molecular responses between the two conditions, we categorized the DEGs into six distinct groups (Figure 1C, File S2, Supporting Information):

1. Common Upregulated Genes: 66 DEGs were upregulated in both OxyHb‐HFA and MetHb‐HFA.
2. OxyHb‐HFA Specific Upregulation: 56 DEGs were upregulated exclusively in OxyHb‐HFA.
3. MetHb‐HFA Specific Upregulation: 44 DEGs were upregulated exclusively in MetHb‐HFA.
4. Common Downregulated Genes: 29 DEGs were downregulated in both conditions.
5. OxyHb‐HFA Specific Downregulation: 59 DEGs were downregulated exclusively in OxyHb‐HFA.
6. MetHb‐HFA Specific Downregulation: 43 DEGs were downregulated exclusively in MetHb‐HFA.

Further enrichment analysis using Gene Ontology (GO) for Biological Processes and Reactome pathways revealed significant enrichment only for the common upregulated and downregulated genes (File S3, Supporting Information). Notably, the commonly upregulated genes in both OxyHb‐HFA and MetHb‐HFA were enriched in pathways related to immune, oxidative stress, and inflammatory responses, suggesting that these processes are critically involved in the cellular response to RBCL exposure (Figure 1D).

We validated these key findings through qPCR analysis, confirming that both OxyHb‐HFA and MetHb‐HFA groups exhibited increased expression of key genes associated with inflammation and oxidative stress, including HMOX1, IL1B, IL6, PTGS2, CCL20, and ICAM1 (Figure 1E). These results underscore the significant impact of RBCL exposure on the inflammatory and stress‐response pathways in HFA.

### 3.3 HBMEC Transcriptome Exploratory Analysis

**Figure 2:**
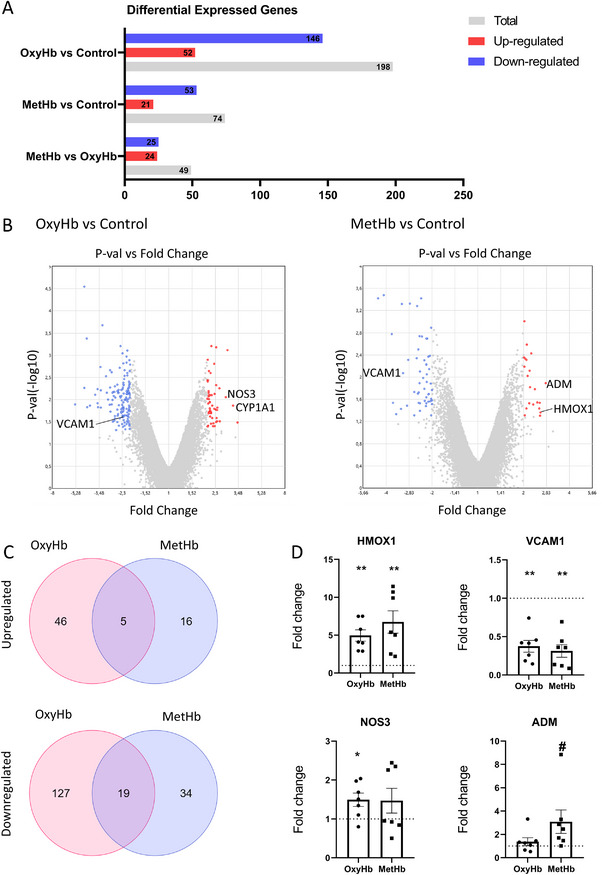
Differential Gene Expression and Functional Interactions in human brain endothelial cells (EC) OxyHb‐EC and MetHb‐EC Conditions Compared to Control‐EC. A) Number of differentially expressed genes (DEGs) in the OxyHb‐EC and MetHb‐EC groups compared to the Control‐EC group. B) Volcano plot depicting the distribution of DEGs between the OxyHb‐EC and MetHb‐EC groups. C) Venn diagrams showing the overlap of upregulated and downregulated DEGs unique to OxyHb, unique to MetHb, and common to both conditions. Created with https://molbiotools.com/. D) Validation of key genes through qPCR analysis. Results were considered statistically significant when the fold change differed from 1 (control level). *p < 0.05, **p < 0.01 (one‐sample Student's *t*‐test); #*p* < 0.05 (Wilcoxon signed‐rank test, used for groups not following a normal distribution as determined by the Shapiro‐Wilk test). Results from 7 independent assays. HMOX1: Heme Oxygenase 1, VCAM1: Vascular Cell Adhesion Molecule 1, NOS3: Nitric Oxide Synthase 3 (Endothelial NOS), ADM: Adrenomedullin, CYP1A1: Cytochrome P450 Family 1 Subfamily A Member 1.

In our exploratory transcriptome analysis of HBMEC exposed to RBCL/OxyHb and RBCL/MetHb, we identified 198 DEGs in the OxyHb‐HBMEC group, with 146 genes upregulated and 52 downregulated. In the Met‐HBMEC group, we found 74 DEGs, comprising 53 upregulated and 21 downregulated genes (**Figure** **2**A, File S4, Supporting Information). The distribution of these DEGs is visually represented in the volcano plots shown in Figure 2B.

To better understand the specific and shared molecular responses between the two conditions, we categorized the DEGs into six distinct groups (Figure 2C, File S5, Supporting Information):

1. Common Upregulated Genes: 5 DEGs were upregulated in both Oxy‐HBMEC and Met‐HBMEC.
2. Oxy‐HBMEC Specific Upregulation: 46 DEGs were upregulated exclusively in Oxy‐HBMEC.
3. Met‐HBMEC Specific Upregulation: 16 DEGs were upregulated exclusively in Met‐HBMEC.
4. Common Downregulated Genes: 19 DEGs were downregulated in both conditions.
5. Oxy‐HBMEC Specific Downregulation: 127 DEGs were downregulated exclusively in Oxy‐HBMEC.
6. Met‐HBMEC Specific Downregulation: 34 DEGs were downregulated exclusively in Met‐HBMEC.

Further enrichment analysis using GO for Biological Processes and Reactome pathways revealed significant enrichment exclusively among the common downregulated genes (File S6, Supporting Information). Notably, many of these downregulated genes were associated with the metallothionein pathway, which plays a crucial role in binding metal ions, particularly cadmium and zinc. This result was unexpected, as existing literature does not clearly support a connection between this pathway and the pathophysiology of the conditions we studied. Given the limited insights from this pathway enrichment, we conducted a manual data exploration, which led to another surprising finding: VCAM1, responsible for leukocyte adhesion and transendothelial migration, was found to be downregulated. Besides that, there were no apparent signs of an inflammatory response in the HBMECs, despite the upregulation of HMOX1, a gene associated with hemoglobin interaction (Figure 2D).

Despite the non‐inflammatory response of endothelial cells to hemoglobin, two observations stood out. NOS3 and adrenomedullin (ADM), both of which are potent vasodilators, were upregulated. Interestingly, we identified a specific transcript of NOS3 using the AceView tool integrated with TAC (File S7, Supporting Information). The upregulation of ADM was confirmed exclusively in the MetHb‐HBMEC group (Figure 2D).

Taken together, the transcriptomic data from HFA and HBMEC indicate that no significant enrichment of critical pathways was observed in response to a specific hemoglobin oxygenation state. We proceeded with experiments involving RBCL/OxyHb.

### 3.4 Assembling the SVZ Chip

**Figure 3:**
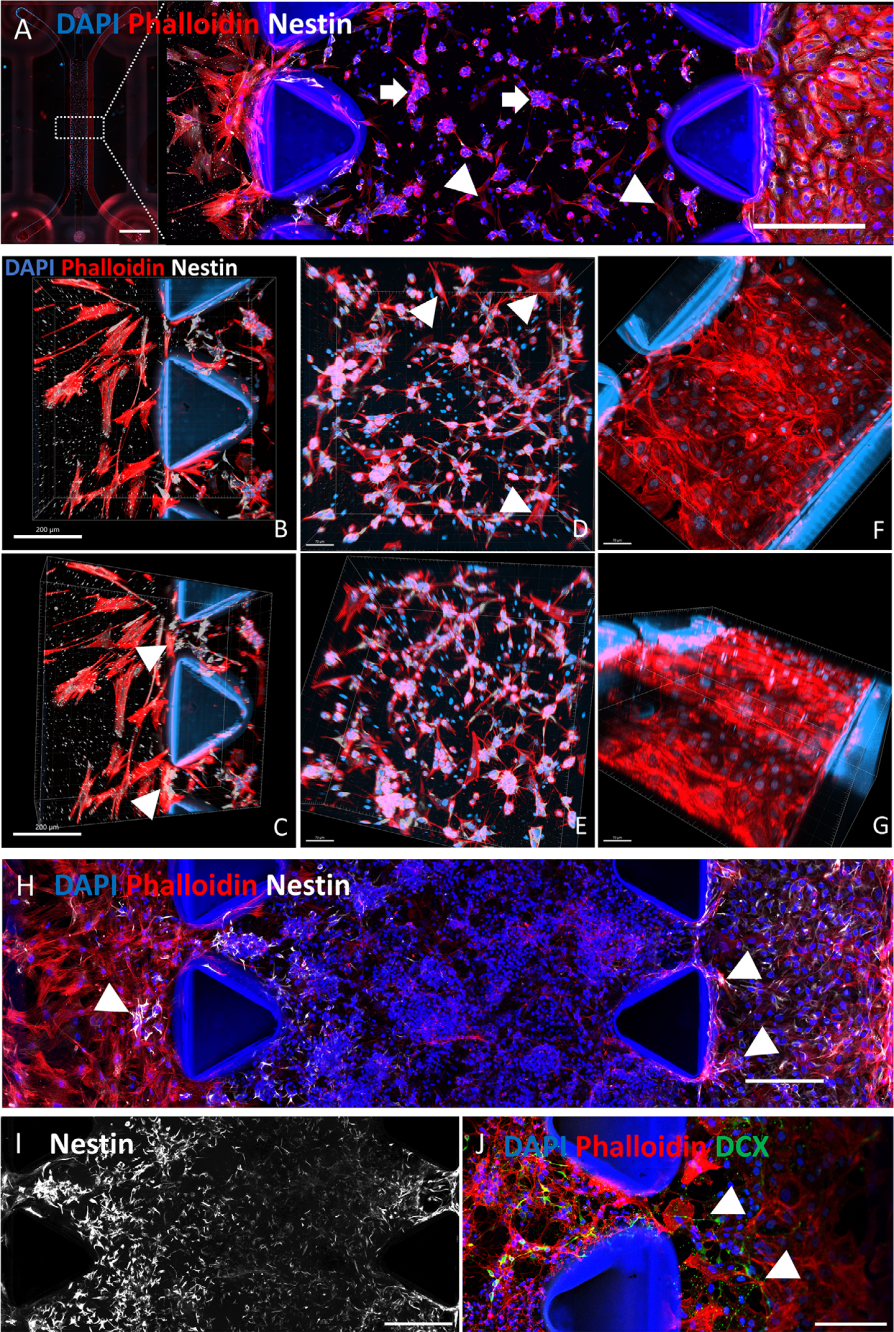
Overview of the SVZ‐on‐a‐chip. A) The SVZ‐on‐a‐chip structure 24 h post‐seeding, showing distinct cellular compartments visualized via staining. The entire chip structure is shown (scale bar = 3000 µm) alongside a magnified maximum Z‐projection view (scale bar = 100 µm). Arrows indicate neural stem cells (NSCs), and triangles highlight human fetal astrocytes (HFAs) embedded in Matrigel, occupying the central channel. The right channel is lined with human brain microvascular endothelial cells (HBMECs), while the left channel contains human choroid plexus epithelial cells (HCPEpiC). B) Neural channel with HCPEpiC seeded along the central channel (scale bar = 200 µm). C) The central channel, highlighted by triangles, is shown in closer detail (scale bar = 200 µm). D) 3D view of the inner channel, showing cells stained with Nestin (gray). Astrocytes, marked by triangles, are stained red but lack Nestin expression (scale bar = 70 µm). E) Inner channel viewed from a different angle (scale bar = 70 µm). F) Vascular channel lined with HBMECs (scale bar = 70 µm). G) HBMECs fully covering the vascular channel (scale bar = 70 µm). H) A magnified maximum Z‐projection view of the SVZ‐on‐a‐chip 7 days after complete seeding, showing significant cellular growth (scale bar = 100 µm). Clusters of NSCs are observed invading both the neural and, predominantly, the vascular channel. I) Maximum Z‐projection view of Nestin staining in the middle channel (scale bar = 100 µm). J) Close‐up of DCX‐positive neural stem cells invading the vascular channel. DCX staining was used instead of Nestin because HBMECs also express Nestin (scale bar = 50 µm). SVZ: Subventricular Zone, NSCs: Neural Stem Cells, HFAs: Human Fetal Astrocytes, HBMECs: Human Brain Microvascular Endothelial Cells, HCPEpiC: Human Choroid Plexus Epithelial Cells, DCX: Doublecortin.

To assess the compartmentalization and cellular organization in the SVZ‐on‐a‐chip model, we monitored the distribution and behavior of cells over time. **Figure** **3**A illustrates the overall structure of the chip 24 h after complete seeding. As defined by the assembly methodology, NSCs and HFAs, embedded in Matrigel, effectively populated the central channel, while HBMECs lined the right channel, and HCPEpiC occupied the left channel. The 3D view of the entire chip and its compartments can be seen in Videos S1 to S5, Supporting Information.

A 3D visualization of the chip compartments is shown in Figure 3B–G, providing a detailed view of cellular organization at the 24‐h mark. The neural channel was populated by HCPEpiCs (Figure 3B), and arrows highlight the cells covering the central channel (Figure 3C). Astrocytes, identifiable by the absence of Nestin staining (gray), were observed within the inner channel in red (Figure 3D), while a different perspective of the same region is shown in Figure 3E. The vascular channel, lined with HBMECs, appeared well‐structured, as shown in Figure 3F, with HBMECs covering the entire vascular channel (Figure 3G).

7 days after complete seeding, the SVZ‐on‐a‐chip demonstrated substantial cellular growth and organization. Clusters of NSCs were observed actively migrating and invading both the neural and vascular channels, with a pronounced preference for the vascular channel (Figure 3H). This migration, however, posed a risk of disrupting the channel architecture and compromising the compartmentalization established within the chip. Consequently, we opted to limit the use of the SVZ‐chip to 7 days post‐seeding. Nestin staining revealed the distribution of NSCs within the middle channel, emphasizing their structural integration (Figure 3I). Furthermore, Doublecortin (DCX)‐positive neuroblasts derived from NSCs were detected in the vascular channel, confirming their migration into this compartment. DCX staining was used instead of Nestin to avoid cross‐reactivity, as HBMECs also express Nestin,^[^ ^4^, ^5^ ^]^ thereby ensuring the specific identification of NSC‐derived neuroblasts (Figure 3J).

### 3.5 SVZ‐on‐a‐Chip Inflammatory Profile in Response to RBCL/OxyHb Exposure

**Figure 4:**
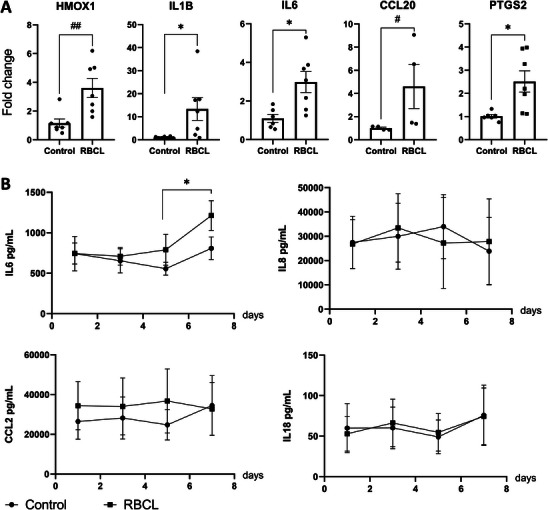
QPCR and Inflammatory Response Analysis in the SVZ‐on‐a‐Chip Model. A) qPCR analysis of selected inflammatory cytokines and related genes. Results from 6 chips control and 7 chips RBCL. Statistically significant differences are indicated as follows: *p < 0.05, **p < 0.01 (Student's *t*‐test); #*p* < 0.05 (Mann‐Whitney test, applied for groups not following a normal distribution as determined by the Shapiro‐Wilk test). Effect size (Cohen's d, 95% CI): HMOX1: 1.80 (0.57 to 3.05), IL1B: 1.34 (0.18 to 2.50), IL6: 1.69 (0.43 to 2.97), CCL20: 1.33 (–0.20 to 2.87), PTGS2: 1.65 (0.39 to 2.91). B) Cytokine levels were measured in a media pool collected from the vascular side of the SVZ‐on‐a‐chip after RBCL/OxyHb exposure. Statistical analysis was applied to the technical quadruplicates. There is an increase in IL6 levels in the RBCL group from day 5 to day 7. * = *p* < 0.05, two‐way Anova. HMOX1: Heme Oxygenase 1, IL1B: Interleukin 1 Beta, IL6: Interleukin 6, CCL20: C‐C Motif Chemokine Ligand 20, PTGS2: Prostaglandin‐Endoperoxide Synthase 2, IL8: Interleukin 8, IL18: Interleukin 18, CCL2: Chemokine (CC motif) ligand 2.

To explore the inflammatory state in the SVZ‐on‐a‐chip model, we first analyzed gene expression profiles on the seventh day post‐exposure to RBCL/OxyHb. Significant upregulation of HMOX1 and several inflammation‐related genes, including IL1B, IL6, CCL20, and PTGS2, was observed (**Figure** **4**A). However, there were no differences in the expression of ICAM, NF‐κβ, CCL2, IL8, or the vasodilators NOS3 and ADM (Figure S4, Supporting Information).

To assess reproducibility and stability, we analyzed the coefficient of variation (CV) across multiple chips exposed to RBCL. In seven independent RBCL‐treated chips versus seven controls, HMOX1 upregulation showed a CV of 49%, whereas IL1B upregulation reached 97%. Although these CV values are relatively high, they were comparable to those observed in astrocytes cultured in Transwells (**Table** **2**), indicating that the variability within the chip model is consistent with that of conventional in vitro systems.

**Table 2:** Coefficient of variation across gene upregulation among chips and transwells exposed to RBCL.

	HMOX1	IL1B	IL6	CCL20	PTGS2
Chip	49	97	48	82	48
TW/HFA/Oxy	29	123	44	89	76
TW/HFA/Met	41	78	73	60	47

HFA: Human Fetal Astrocytes, RBCL: red blood cell lysates, HMOX1: Heme Oxygenase 1, IL1B: Interleukin 1 Beta, IL6: Interleukin 6, CCL20: C‐C Motif Chemokine Ligand 20, PTGS2: Prostaglandin‐Endoperoxide Synthase 2.

Following the gene expression analysis, we evaluated the cytokine profile in the vascular channel using a multiplex assay. Four cytokines—IL‐8, IL‐6, CCL2, and IL‐18 were detected at measurable levels above the assay's threshold (Figure 4B). IL‐6 exhibited a distinct temporal pattern, with levels progressively increasing on the fifth and seventh days post RBCL exposure. By the seventh day, IL‐6 concentrations reached ≈1200 pg mL^−1^, compared to ≈745 pg mL^−1^ on the first day, while levels in the control group remained stable at ≈800 pg mL^−1^ (Figure 4A). The cytokine levels in the vascular channel were markedly different from those detected in the RBCL (neural channel), confirming that the secreted cytokines originated from the cells comprising the SVZ‐on‐a‐chip (**Table** **3**).

**Table 3:** Levels of Inflammatory Cytokines in CSF and RBCL/ OxyHb Samples.

Cytokine (pg mL^−1^)	RBCL/OxyHb 10 %	Highest value measured in the vascular channel after RBCL exposure	CSF	Highest value measured in the vascular channel after CSF exposure
IL1B	0	0	30.98	0
IFNα2	0	0	1.98	0
IFNγ	0	0	23.75	0
TNFα	0	0	31.90	0
CCL2 (MCP1)	23.02	36 761.30	11.91	29 452.20
IL6	0	1212.82	663.45	921.45
IL8	1.31	33 421.80	9616.25	39 887.60
IL10	0	0	27.85	0
IL12p70	0	0	2.14	0
IL17A	0	0	0.40	0
IL18	134.86	74.44	134.57	42.72
IL23	0	0	1.64	0
IL33	4.08	0	0	0

IL1B: Interleukin1 beta, IFNα2: Interferon alpha 2, IFNγ: Interferon gamma, TNFα: Tumor Necrosis Factor alpha, CCL2 (MCP1): Chemokine (CC motif) ligand 2 (Monocyte Chemoattractant Protein1), IL6: Interleukin6, IL8: Interleukin8, IL10: Interleukin10, IL12p70: Interleukin12, p70, IL17A: Interleukin17A, IL18: Interleukin18, IL23: Interleukin23, IL33: Interleukin33.

### 3.6 Neurosphere Assay

**Figure 5:**
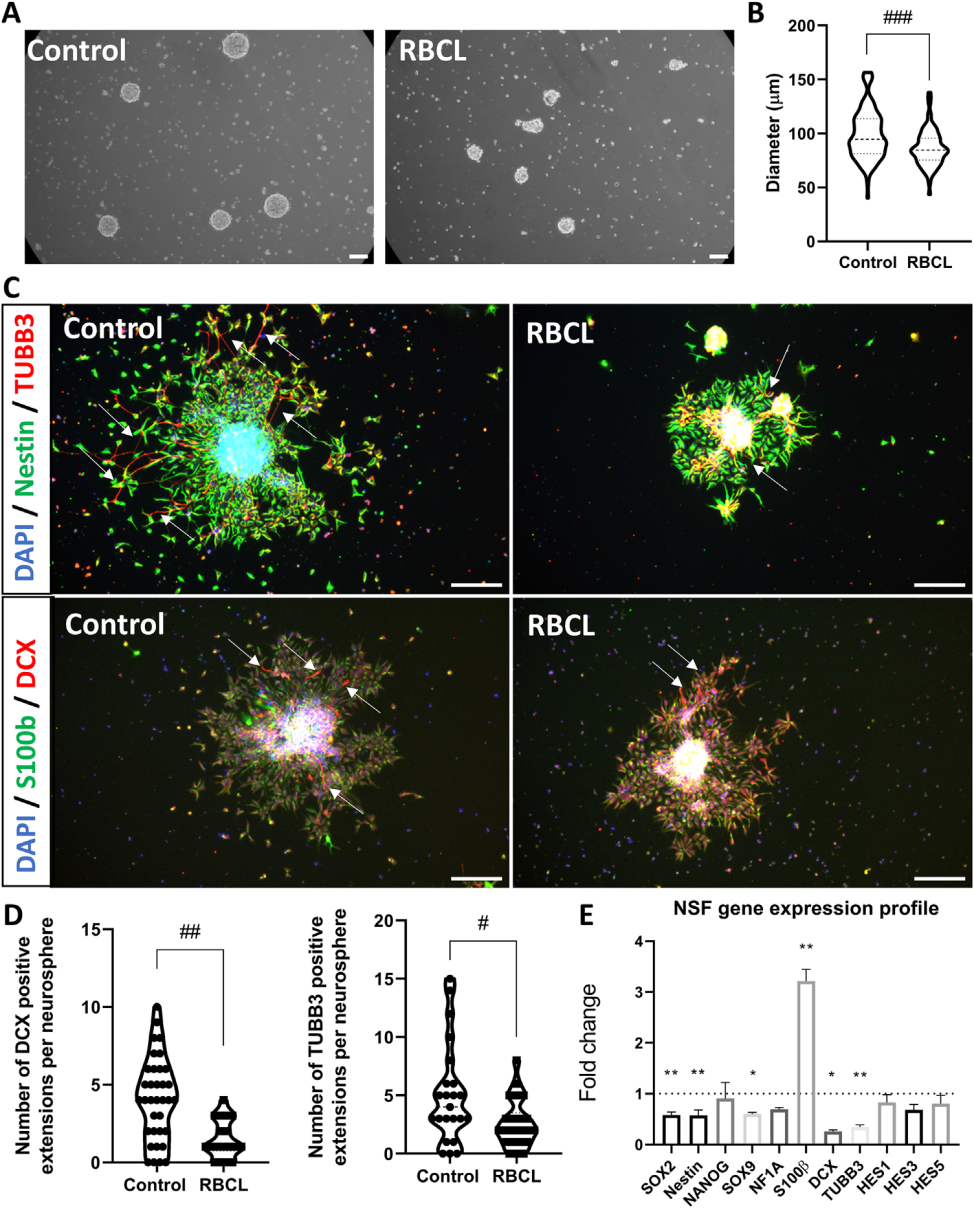
Impact of RBCL Exposure on Neural Stem Cell Behavior Assessed by Neurosphere Assay. A) Representative images of neurospheres (NSF) formed from neural stem cells (NSCs) after 6 days in culture under floating conditions. Scale bar = 100 µm. B) Quantitative analysis of neurosphere diameters, showing a significant reduction in NSCs exposed to red cell blood lysate (RBCL) compared to the control group. ### = *p* < 0.001 (N= 107 NSF control and 82 NSF RBCL, Mann‐Whitney test, applied for groups not following a normal distribution as determined by the Shapiro‐Wilk test). Effect size (Cohen's d, 95% CI) −0.627 (−0.922 to −0.333). C) Representative images of neurite outgrowth (arrows) from NSCs seeded onto PLO‐Laminin‐coated wells and cultured without growth factors for 48 h, stained for DCX and TUBB3. Scale bar = 200 µm. D) Quantification of neurite outgrowth, revealing fewer DCX and TUBB3‐positive neurites in the RBCL‐exposed group. # = *p* < 0.05 and *## p* < 0.01 (DCX, N= 35 NSF control and 18 NSF RBCL, TUBB3, N= 24 NSF control and 22 NSF RBCL, Mann‐Whitney test, applied for groups not following a normal distribution as determined by the Shapiro‐Wilk test). Effect size DCX (Cohen's d, 95% CI) −1.131 (−1.739 to −0.523), Effect size TUBB3 (Cohen's d, 95% CI) ‐ −0.715 (−1.318 to −0.112), E) Gene expression analysis showing decreased levels of SOX2, Nestin, SOX9, DCX, and TUBB3, along with increased S100β expression in NSCs exposed to RBCL. Results from one RNA pool, with statistical tests applied to 6 technical replicates (except NF1A, DCX, and S100β =3 technical replicates). * = *p* < 0.05 and ** = *p* < 0.01, Student's t‐test. SOX2: SRY‐Box Transcription Factor 2, Nestin: Nestin (Neural Stem Cell Marker), NANOG: Nanog Homeobox, SOX9: SRY‐Box Transcription Factor 9, NFIA: Nuclear Factor I A, S100β: S100 Calcium Binding Protein B, DCX: Doublecortin, TUBB3: Tubulin Beta 3 Class III, HES1: Hes Family BHLH Transcription Factor 1, HES3: Hes Family BHLH Transcription Factor 3, HES5: Hes Family BHLH Transcription Factor 5, DAPI: 4′,6‐diamidino‐2‐phenylindole.

The neurosphere (NSF) assay is a well‐established method to assess NSC proliferation and self‐renewal. To examine NSC behavior following IVH, we dissociated the extracellular matrix and cultured NSCs under floating conditions in expansion media. After 6 days, the diameters of the resulting neurospheres were measured. Because cells were counted and seeded at equal density into the AggreWell, neurosphere size reflects the proliferation rate of NSCs rather than differences in cell number. The culture conditions, together with the higher proliferative capacity of NSCs, favor their enrichment within the spheres. In line with this, the neurospheres did not express markers of astrocytes (GFAP), endothelial cells (PECAM‐1), or choroid plexus (transthyretin) (Figure S5, Supporting Information). NSCs exposed to RBCL generated significantly smaller neurospheres than controls (**Figure** **5**A,B; Mann–Whitney test, p < 0.001; Cohen's d = –0.627, 95% CI –0.922 to –0.333). When plated onto poly‐Laminin–coated wells and cultured without growth factors for 48 h, RBCL‐exposed neurospheres produced fewer DCX‐ and TUBB3‐positive neurites (Figure 5C,D), confirmed by quantitative analysis (DCX: Cohen's d = –1.131, 95% CI –1.739 to –0.523; TUBB3: Cohen's d = –0.715, 95% CI –1.318 to –0.112). Consistently, gene expression analysis revealed downregulation of SOX2, Nestin, SOX9, DCX, and TUBB3, accompanied by increased expression of S100β (Figure 5E). Together, these findings suggest that RBCL exposure impairs NSC proliferation and neuronal differentiation, while promoting glial lineage commitment.

### 3.7 Comparative Inflammatory Response to Newborn CSF Exposure

**Figure 6:**
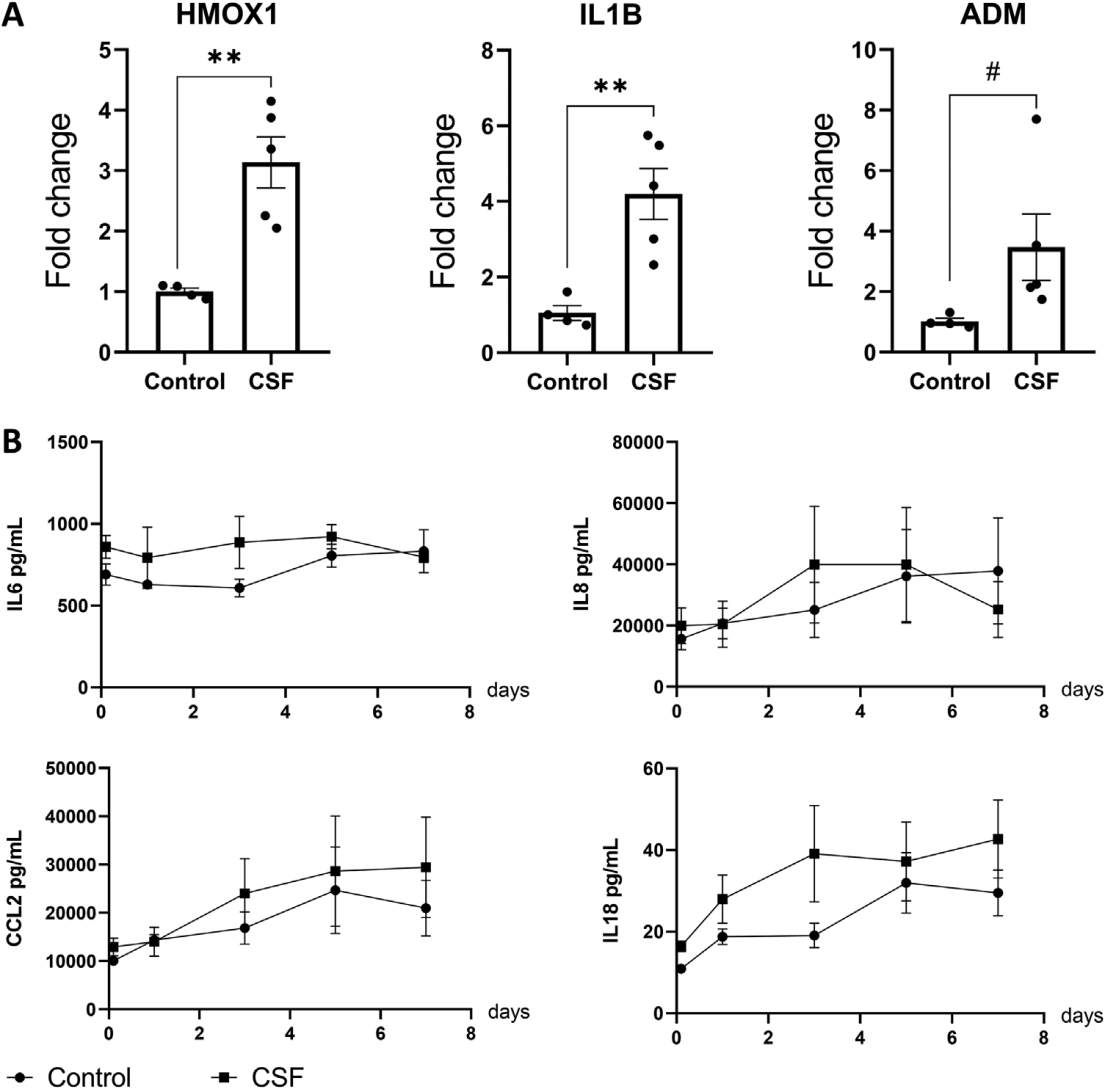
Inflammatory Response to the CSF in the he SVZ‐on‐a‐Chip Model. A) qPCR analysis of selected inflammatory cytokines and related genes. N 4 chips control and 5 CSF. Statistically significant differences are indicated as follows: **p < 0.01 (Student's *t*‐test); #*p* < 0.05 (Mann‐Whitney test, applied for groups not following a normal distribution as determined by the Shapiro‐Wilk test). Effect size (Cohen's d, 95% CI): HMOX1: 2.989 (1.083 to 4.896), IL1B: 2.707 (0.893 to 4.522), ADM: 1.322 (−0.128 to 2.772). B) Cytokine levels were measured in a media pool collected from the vascular side of the SVZ‐on‐a‐chip after exposure to CSF. We did not observe a clear increase in any of the cytokines evaluated. Statistical analysis was applied to the technical quadruplicates. HMOX1: Heme Oxygenase 1, IL1B: Interleukin 1 Beta, ADM: adrenomedullin, IL8: Interleukin 8, IL18: Interleukin 18, CCL2: Chemokine (CC motif) ligand 2.

When the SVZ‐on‐a‐chip received the hemorrhagic CSF from preterm infants in the neural chamber, the inflammatory response was notably milder compared to RBCL/OxyHb exposure. qPCR analysis revealed a generally lower upregulation of inflammatory genes, with only IL1B showing significant increases (**Figure** **6**A). Additionally, HMOX1 was also found to be upregulated. Interestingly, ADM, which had been upregulated in response to RBCL/ MetHb in HBMECs, was also upregulated following CSF exposure (Figure 6A). Genes with no differential expression response are shown in Figure S6, Supporting Information.

Consistent with the gene expression data, the cytokine profile showed no significant increase in the levels of the evaluated cytokines, including IL6 (Figure 6B).

### 3.8 Effects of Hemorrhagic CSF Exposure on Neurospheres

**Figure 7:**
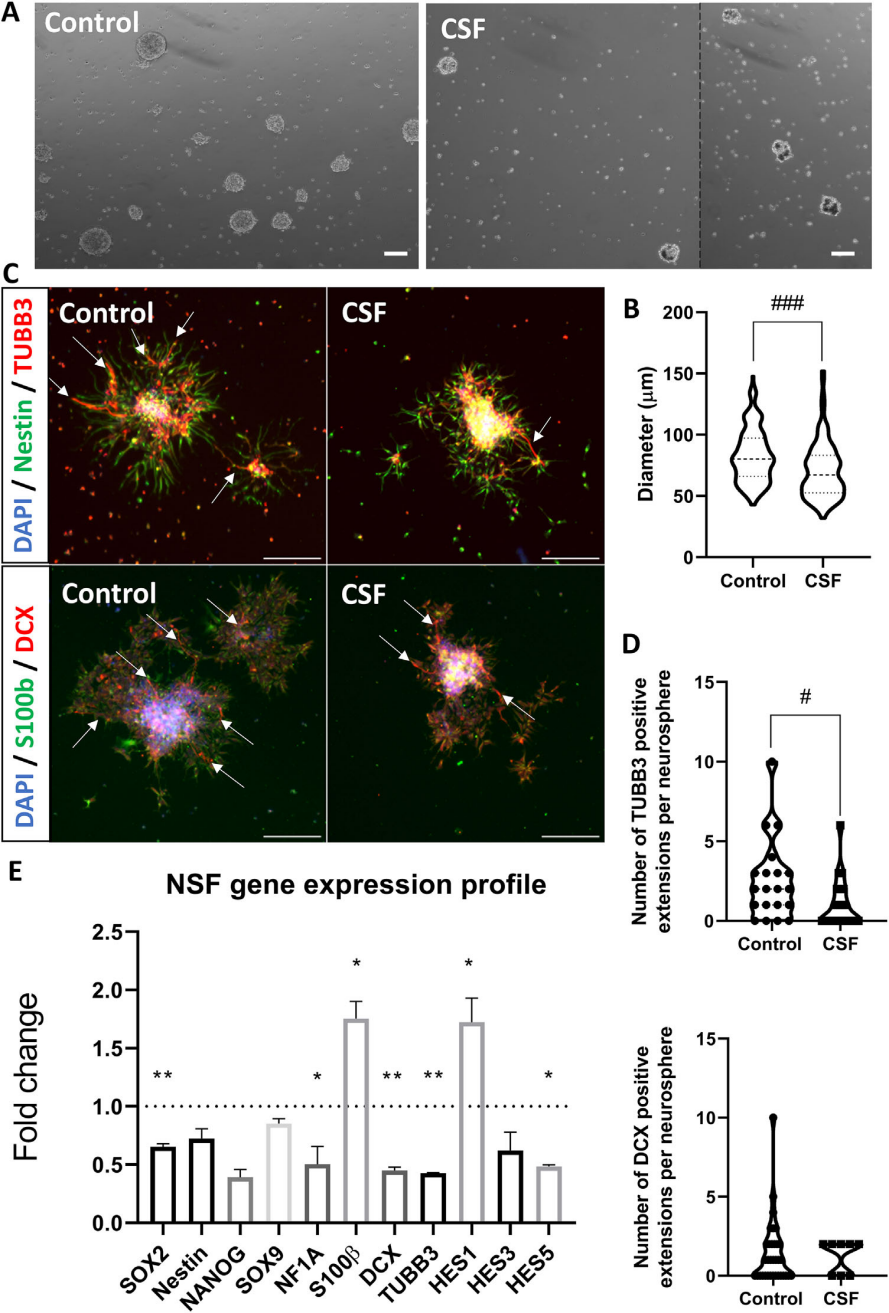
Impact of CSF Exposure on Neural Stem Cell Behavior Assessed by Neurosphere Assay. A) Representative images of neurospheres (NSF) formed from neural stem cells (NSCs) after 6 days in culture under floating conditions. Scale bar = 100 µm. A dashed line indicates where two microscopic fields were merged, as no single field contained a sufficient number of neurospheres to provide a representative view. The corresponding raw images are shown in Figure S7, Supporting Information. B) Quantitative analysis of neurosphere diameters, showing a significant reduction in NSCs exposed to the CSF compared to the control group. ### = *p* < 0.001 (N= 151 NSF controls and 110 NSF CSF, Mann‐Whitney test, applied for groups not following a normal distribution as determined by the Shapiro‐Wilk test). Effect size (Cohen's d, 95% CI) −0.548 (−0.798 to −0.298). C) Representative images of neurite outgrowth (arrows) from NSCs seeded onto PLO‐Laminin‐coated wells and cultured without growth factors for 48 h, stained for DCX and TUBB3. Scale bar = 200 µm. D) Quantification of neurite outgrowth, revealing fewer TUBB3‐positive neurites in the CSF‐exposed group. # = *p* < 0.05 ((DCX, N= 34 NSF control and 8 NSF RBCL, TUBB3, N= 20 NSF control and 22 NSF RBCL) Mann‐Whitney test, applied for groups not following a normal distribution as determined by the Shapiro‐Wilk test). Effect size DCX (Cohen's d, 95% CI) −0.07 (−0.841 to 0.7). Effect size TUBB3 (Cohen's d, 95% CI) −73 (−1.362 to −0.111). E) Gene expression analysis showing decreased levels of SOX2, NF1A, DCX, and TUBB3, along with increased S100β and HES1 expression and reduced HES5 expression, in NSCs exposed to CSF. Results from an RNA pool, with statistical tests applied to 3 technical replicates. *= *p* < 0.05 and ** = *p* < 0.01, Student's *t*‐test. SOX2: SRY‐Box Transcription Factor 2, Nestin: Nestin (Neural Stem Cell Marker), NANOG: Nanog Homeobox, SOX9: SRY‐Box Transcription Factor 9, NFIA: Nuclear Factor I A, S100β: S100 Calcium Binding Protein B, DCX: Doublecortin, TUBB3: Tubulin Beta 3 Class III, HES1: Hes Family BHLH Transcription Factor 1, HES3: Hes Family BHLH Transcription Factor 3, HES5: Hes Family BHLH Transcription Factor 5, DAPI: 4′,6‐diamidino‐2‐phenylindole.

In a parallel set of experiments, we assessed the effects of hemorrhagic CSF on NSC behavior using the neurosphere assay. Compared with controls, CSF‐exposed NSCs generated significantly smaller neurospheres (**Figure** **7**A,B; Mann–Whitney test, p < 0.001; Cohen's d = –0.548, 95% CI –0.798 to –0.298), indicating impaired proliferative capacity in suspension. When plated onto poly‐Laminin–coated wells and cultured without growth factors, these neurospheres exhibited a marked reduction in TUBB3‐positive neurite formation, while DCX‐positive neurite outgrowth showed no significant change (Figure 7C,D; TUBB3: Cohen's d = –0.736, 95% CI –1.362 to –0.111; DCX: Cohen's d = –0.07, 95% CI –0.841 to 0.700). At the transcriptional level, CSF exposure resulted in the downregulation of SOX2, NFIA, DCX, and TUBB3, accompanied by increased expression of S100β and HES1, and reduced expression of HES5 (Figure 7E). Together, these results suggest that hemorrhagic CSF compromises NSC proliferation and neuronal differentiation, while promoting a shift toward glial lineage commitment and perturbing Notch signaling pathways.

### 3.9 Oxidative Stress Detection in Neurospheres

**Figure 8:**
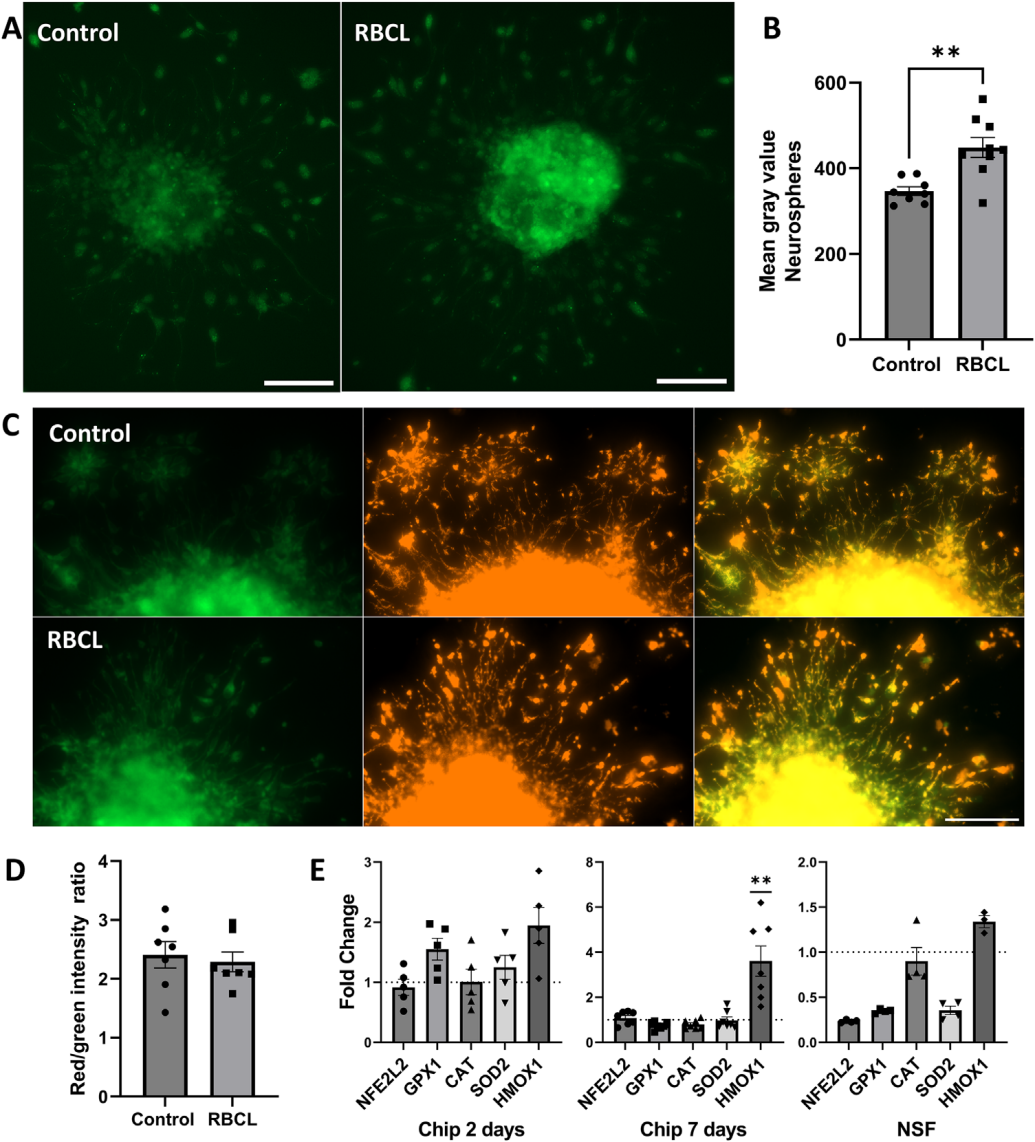
Analysis of oxidative stress in neurospheres. A) Increased production of reactive oxygen species (ROS) in neurospheres exposed to RBCL. Scale bar = 100 µm. B) Quantification of mean gray values in CellROX‐treated neurospheres (**p < 0.01, Student's *t*‐test; *n* = 8 for control and *n* = 9 for RBCL). Effect size (Cohen's d, 95% CI): 1.859 (0.72 to 2.998). C) JC‐1 staining of neurospheres showing no evidence of mitochondrial dysfunction. Scale bar = 100 µm. D) Quantification of the red/green fluorescence intensity ratio in JC‐1‐stained neurospheres (*n* = 7). E) Gene expression analysis of oxidative stress–related genes in chips (2 days, n=5, and 7 days, n=7) and in neurospheres (results from an RNA pool, with statistical tests applied to 4 technical replicates). **p < 0.01 (Student's *t*‐test). RBCL: red blood cell lysate; NFE2L2: nuclear factor erythroid 2–related factor 2; GPX1: glutathione peroxidase 1; CAT: catalase; SOD2: superoxide dismutase 2; HMOX1: heme oxygenase 1.

The next step was to investigate potential mechanisms underlying the reduced neurosphere size and impaired neurogenesis observed in chips exposed to RBCL. We first assessed the role of oxidative stress. Neurospheres from chips exposed to RBCL exhibited a significant increase in reactive oxygen species (ROS) production, as assessed by the CellROX assay, confirming the induction of oxidative stress (**Figure** **8**A,B). Despite this increase, no alterations in mitochondrial function were detected (Figure 8C,D), suggesting that oxidative damage did not compromise the integrity or bioenergetic activity of the organelles. Gene expression analysis of NFE2L2, GPX1, CAT, and SOD2 showed no significant differences between groups, either in the SVZ‐on‐a‐chip model at days 2 and 7 or in isolated neurospheres, indicating the absence of activation of the antioxidant response under RBCL‐induced oxidative stress (Figure 8E). Taken together, these findings suggest that although ROS levels are elevated, the major antioxidant defense pathways are not upregulated in response to this increase.

### 3.10 Effects of IL1 Receptor Antagonist on Neurogenesis

In parallel to oxidative stress analysis, given that our data indicate IL1B‐mediated pathways are consistently upregulated under both RBCL and hemorrhagic CSF conditions—and considering previous reports of IL1B's inhibitory effects on neurogenesis—we directly tested this hypothesis by evaluating NSC behavior in chips treated with an interleukin‐1 receptor antagonist (IL1RA). While IL1RA reported effective concentrations range from 5 to 100 ng mL^−1^ (typically 20–40 ng mL^−1^), we used 100 ng mL^−1^ to ensure complete receptor blockade.^[^ ^27^, ^28^ ^]^

**Figure 9:**
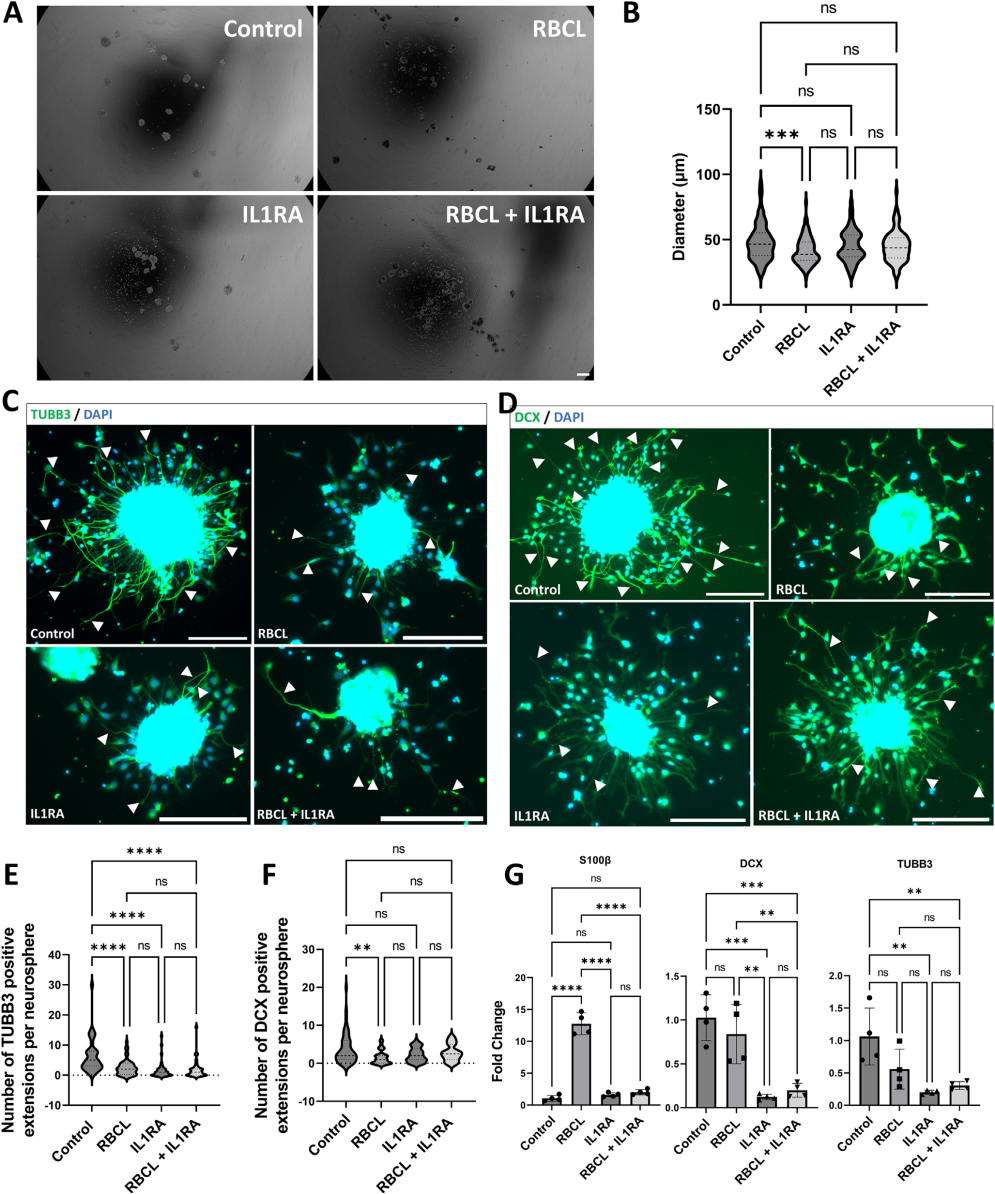
Impact of IL1RA on the behavior of neural stem cells (NSCs) exposed to RBCL, assessed by the neurosphere assay. A) Representative images of neurospheres (NSFs) formed from NSCs after 6 days of suspension culture. Scale bar = 100 µm. B) Quantitative analysis of neurosphere diameters showing a significant reduction in NSCs exposed to RBCL compared to the control group (***p < 0.001, one‐way ANOVA). Effect size from Control to RBCL (Cohen's d, 95% CI) −0.534 (−0.812 to −0.256), Effect size from RBCL to RBCL + IL1RA (Cohen's d, 95% CI) 0.309(0.025 to 0.594). C) Representative images of neurite outgrowth (arrows) from NSCs plated on PLO‐Laminin–coated wells and cultured without growth factors for 48 h, stained for TUBB3. Scale bar = 200 µm. D) Representative images of neurite outgrowth (arrows) from NSCs plated on PLO‐Laminin–coated wells and cultured without growth factors for 48 h, stained for DCX. Scale bar = 200 µm. E) Quantification of neurite outgrowth showing a reduced number of TUBB3‐positive neurites in all experimental groups compared to control (****p < 0.001, one‐way ANOVA). Effect size from Control to RBCL (Cohen's d, 95% CI) −0.869 (−1.311 to −0.427). F) Quantification of neurite outgrowth showing a reduced number of DCX‐positive neurites in the RBCL‐exposed group (**p < 0.01, one‐way ANOVA). Effect size from Control to RBCL (Cohen's d, 95% CI) −0.664 (−1.099 to −0.229), Effect size from RBCL to RBCL + IL1RA (Cohen's d, 95% CI) 0.606 (0.107 ‐ 1.105). G) Gene expression analysis showing reduced DCX and TUBB3 levels in groups treated with IL1RA. An increase in S100β expression was observed in NSFs exposed to RBCL, whereas IL1RA‐treated NSFs expressed S100β at levels comparable to control. Data were obtained from pooled RNA, with statistical tests applied to technical replicates. **p < 0.01, ***p < 0.001, ****p < 0.0001 (one‐way ANOVA). RBCL: red blood cell lysate; IL1RA: interleukin‐1 receptor antagonist; S100β: S100 calcium‐binding protein β; DCX: doublecortin; TUBB3: class III β‐tubulin; DAPI: 4′,6‐diamidino‐2‐phenylindole.

**Figure** **9** demonstrates that exposure of NSCs to RBCL markedly impaired their neurogenic potential, as reflected by the significant reduction in neurosphere diameter and neurite outgrowth (Figure 9A,B). The effect sizes confirmed a moderate to large detrimental impact of RBCL on neurosphere formation (Cohen's d = −0.534) and on neurite extension, particularly for TUBB3‐ and DCX‐positive cells (Cohen's d = −0.869 and −0.664, respectively) (Figure 9C–F). Treatment with IL1RA partially mitigated these effects, leading to modest improvements in neurosphere size (Cohen's d = 0.309) and a more pronounced recovery of DCX‐positive neurites (Cohen's d = 0.606) (Figure 9D,F), although TUBB3‐positive neurite formation remained impaired (Figure 9C,E). At the transcriptional level, RBCL exposure reduced the expression of DCX and TUBB3, while inducing a glial shift, as evidenced by increased S100β (Figure 9G). Importantly, IL1RA treatment normalized S100β levels to those of the control group, suggesting a selective role in modulating glial differentiation rather than fully restoring neuronal gene expression. Together, these findings indicate that IL1RA provides partial neuroprotection against RBCL‐induced damage in NSCs, with stronger effects on preventing the glial shift.

## 4 Discussion

IVH is a serious complication in preterm newborns that occurs during a critical period of brain development.^[^ ^12^ ^]^ Severe IVH in extremely preterm infants can lead to a range of neurological, sensory, cognitive, and motor disabilities, both in the short and long term.^[^ ^29^ ^]^ Although the association between IVH and neurodevelopmental impairment is well‐established, the exact mechanisms are not yet fully understood.^[^ ^30^ ^]^

Disruption of the SVZ has been identified as a key feature of IVH in human preterms.^[^ ^31^ ^]^ Therefore, it is crucial to understand how blood affects the ventricular zone to develop effective treatments that reduce the neurological sequelae of IVH. An in vitro model of IVH is essential for investigating the impact of blood and its components on the developing SVZ and its stem cell niche.^[^ ^31^ ^]^ In this study, we introduce a novel human SVZ‐on‐a‐chip model integrating primary human cells and iPSC‐derived NSCs into a 3D microenvironment.

Although previous studies have described the importance of red blood cells (RBCs) and hemoglobin metabolites in the development of damage to the immature brain following IVH,^[^ ^32^, ^33^, ^34^, ^35^ ^]^ there is a need to further elucidate the molecular changes induced by RBCs in the neurogenic niche. Inflammation is a major pathological feature associated with IVH, and there is growing evidence that early anti‐inflammatory interventions could be crucial in protecting the fragile, developing white matter in these infants.^[^ ^12^ ^]^ Furthermore, proinflammatory conditions at birth are linked to poor functional outcomes at 2 years of corrected age in children born very preterm.^[^ ^36^ ^]^ Therefore, understanding the inflammatory changes is essential for understanding alterations in the SVZ microenvironment and the behavior of NSCs.

Transcriptomic analysis of primary HFA revealed a notable increase in several inflammatory pathways. Key molecular targets identified include HMOX1, IL1B, IL6, CCL20, PTGS2, and ICAM1. Here, there was no significant difference between the effects of OxyHb and MetHb exposure on these inflammatory pathways, despite the anticipated distinct responses due to the different oxidative states of these hemoglobin derivatives.^[^ ^26^ ^]^

In primary HBMEC, however, transcriptomic analysis revealed a minimal inflammatory response, despite the upregulation of HMOX1. Unexpectedly, we observed a downregulation of VCAM1, which is critical for recruiting peripheral white blood cells to the brain parenchyma.^[^ ^37^, ^38^ ^]^ This finding was counterintuitive, as we expected an upregulation of VCAM1 in response to inflammatory signals, particularly in conditions of vascular injury.^[^ ^38^ ^]^ Interestingly, the upregulation of vasodilatory factors, such as NOS3 (endothelial nitric oxide synthase) in OxyHb‐exposed endothelial cells and ADM (adrenomedullin) in MetHb‐exposed endothelial cells, may indicate vascular dysfunction.

The SVZ‐on‐a‐chip model was constructed with a compartmentalized structure, integrating four cell types to replicate the SVZ environment. The model includes:

‐ A right vascular channel lined with HBMEC.

‐ A central channel containing NSCs and HFAs embedded in a 3D matrix (Matrigel).

‐ A left neural channel with HCPepiC.

Within 48 h, the model begins to exhibit the intended structural organization. Over a 7‐day cultivation period, we observed migration of the NSCs toward the vascular channel, culminating in their invasion of this channel. This migration mimics the natural in vivo movement of NSCs, which typically migrate from the SVZ through the rostral migratory stream (RMS) along blood vessels.^[^ ^39^ ^]^ However, the invasion of the vascular channel by the NSCs ultimately led to the destruction of the channel and loss of compartmentalization, which limited the analysis of the model to 7 days.

When exposing the SVZ‐on‐a‐chip to OxyHb‐RBCL, we observed that the gene expression patterns within the chip mirrored those seen in HFAs, with increased expression of HMOX1, IL1B, IL6, CCL20, and PTGS2. In the secretome analysis, we detected only IL6 in the conditioned media, with levels ≈50% higher in the RBCL group at 7 days.

In contrast, when the SVZ‐on‐a‐chip was exposed to hemorrhagic CSF from preterm infants with IVH, we only noted upregulation of HMOX1 and IL1B at the gene expression level, with no significant increase in cytokine levels in the conditioned media. Interestingly, ADM was again upregulated by CSF, recapitulating the result obtained in RBCL/MetHb‐exposed HBMEC.

To analyze the gene expression responses of NSCs in isolation, we incorporated a neurosphere assay into the chip model. This assay is a cost‐effective alternative to single‐cell sequencing, allowing for the broader application of the model.^[^ ^40^ ^]^

Despite differences in the inflammatory signatures between OxyHb and CSF exposures, NSCs exhibited a consistent response across conditions, characterized by reduced neurosphere diameter, impaired neurite formation (as indicated by DCX and TUBB3 Positivity), and downregulation of SOX2, DCX, and TUBB3, accompanied by S100B upregulation, marking a shift toward glial differentiation. RBCL exposure further induced a marked increase in reactive oxygen species within neurospheres, confirming oxidative stress. However, mitochondrial function remained preserved, and canonical antioxidant pathways were not activated, suggesting that NSCs tolerate acute oxidative challenges without engaging compensatory defenses.

The convergence of NSC responses in both RBCL/OxyHb and hemorrhagic CSF exposures suggests that a common mechanism may drive the observed impairment of neurogenesis. Our data indicate that IL1B, which was upregulated in both conditions, is a key contributor. This is consistent with prior studies showing that IL1B suppresses neurogenesis in human hippocampal progenitor cells ^[^ ^41^ ^]^ and that neonatal exposure to IL1B in mice induces long‐lasting behavioral changes, including anxiety‐like phenotypes and spatial memory deficits.^[^ ^42^ ^]^ In vitro, rat neural progenitor cells exposed to IL1B exhibited reduced neuronal output and increased astrocytic differentiation, effects that were mitigated by IL1 receptor antagonist treatment.^[^ ^43^ ^]^ These findings, which align with our results, suggest that IL1B has both anti‐neurogenic and pro‐gliogenic effects in the developing brain.

Notably, IL1RA treatment only partially restored NSC behavior in our system, suggesting that while IL1B signaling is a critical component, additional inflammatory mediators are likely involved. Our data, therefore, support the interpretation that IL1B contributes significantly to the shift toward gliogenesis observed after hemorrhagic injury.

It is important to emphasize that the primary objective of this study was not to dissect IL1B signaling per se, but rather to establish a robust **human SVZ‐on‐a‐chip model** to investigate mechanisms of neonatal intraventricular hemorrhage. The model successfully reproduced hallmark features of the SVZ niche response to hemorrhagic injury, including reduced neurosphere growth, impaired neurite outgrowth, altered gene expression, and inflammation‐driven gliogenic shift. By mimicking the human neurogenic microenvironment in a controlled setting, the SVZ‐on‐a‐chip provides a powerful platform for studying the cellular and molecular pathways underlying impaired neurogenesis in IVH.

While the system cannot fully recapitulate the complexity of in vivo conditions—including systemic immune interactions, vascular dynamics, and the full spectrum of inflammatory responses—it nonetheless provides a physiologically relevant and reproducible model. Future refinements, such as incorporating additional cell types or dynamic perfusion systems, could further enhance its translational value.

Beyond its value as a mechanistic platform, the SVZ‐on‐a‐chip also holds considerable translational potential. By incorporating patient‐derived iPSCs and cerebrospinal fluid, the system could be tailored to capture individual variability in neurogenic responses, thereby advancing personalized approaches to neonatal brain injury. Such adaptability would enable the identification of patient‐specific susceptibilities and therapeutic windows. In addition, the model offers a physiologically relevant environment for preclinical drug discovery, providing a human‐based platform to evaluate anti‐inflammatory or neuroprotective interventions before clinical translation. Together, these features position the SVZ‐on‐a‐chip as both a powerful research tool and a promising bridge between basic science, precision medicine, and therapeutic development in IVH.

## 5 Conclusions

By simulating IVH within the SVZ‐on‐a‐chip, we describe a model that can enable further understanding of the cellular and molecular mechanisms underlying impaired neurogenesis observed in preterm infants following IVH. Our findings in this study highlight the importance of the inflammatory microenvironment in regulating neurogenesis within the SVZ, with IL1B emerging as a key mediator of this process. Altogether, this innovative tool could significantly enhance our understanding of IVH‐related pathology and facilitate the identification of potential therapeutic targets.

## Conflict of Interest

The authors declare no conflict of interest.

## Author Contributions

L.N.Z.: conceptualized the study, performed the experiments and the analyses, wrote the original draft, and reviewed and edited the document table and figures; B.G.: performed the experiments, reviewed and edited the document table and figures; M.G.: advised the execution of the experiments and analyses, and reviewed and edited the final document; A.S.: advised the execution of the experiments and analyses, and reviewed the final document; M.A.P.: advised the execution of the experiments and analyses, and reviewed and edited the final document; A.H.: conceptualized the study, advised the execution of the experiments and analyses, reviewed and edited the final document. All authors have read and agreed to the final version of the manuscript.

## Supporting information



Supporting Information

Supplemental Figure 1‐7

Supplemental Video 1‐5

Supplemental Table 1

Supplemental Table 2

Supplemental DataFile

## Data Availability

The transcriptomes analyzed in the current study are available on Figshare (10.6084/m9.figshare.28259441 and 10.6084/m9.figshare.28259408). All other data are available upon request by contacting anna.herland@ki.se.
