# Supplementary material for: Subventricular Zone‐on‐a‐Chip: A Model to Study Neurogenesis Disruption in Neonatal Intraventricular Hemorrhage
Source: Adv Sci (Weinh). 2025 Oct 24;13(3):e02145. doi: 10.1002/advs.202502145 (PMC12806495; doi:10.1002/advs.202502145)
Supplement: Supplementary file 1 — Supporting Information [file ADVS-13-e02145-s001.docx]

**Supplementary Figures legend:**

**Supplementary Figure 1: Characterization of Hemoglobin Preparations and Co-culture System.** (A) Spectra of OxyHb and MetHb-RBCL. (B) Comparison of color differences between OxyHb and MetHb preparations, showing the typical red hue of OxyHb compared to the brownish tint of MetHb. (C) Schematic drawing of the co-culture of human brain microvascular endothelial cells (HBMEC) with human fetal astrocytes (HFA) in attachment factor-coated Transwells (TW). Created with Biorender.com.

**Supplementary Figure 2: Flowchart of the transcriptome analysis process.** The flowchart illustrates the steps involved in the analysis, including: (1) comparing transcriptomes to generate lists of differentially expressed genes (DEGs), (2) comparing DEG lists between conditions, and (3) performing gene enrichment analysis.

**Supplementary Figure 3. Schematic representation of the assembly for the SVZ-on-a-chip model.**

**Supplementary Figure 4: Complementary Inflammatory Response Analysis in the SVZ-on-a-Chip Model**. This figure presents the qPCR analysis of cytokines that did not show statistically significant changes in expression levels following RBCL/OxyHb exposure in the SVZ-on-a-chip model. IL8: Interleukin 8, CCL2: C-C Motif Chemokine Ligand 2, ICAM1: Intercellular Adhesion Molecule 1, NF-κβ: Nuclear Factor Kappa B, NOS3: Nitric Oxide Synthase 3 (Endothelial NOS), ADM: Adrenomedullin.

**Supplementary Figure 5: Characterization of Sphere Cell Type Composition.** (A) Gene expression curves show no expression of GFAP (astrocyte marker) or (B) PECAM1 (endothelial marker) in the spheres. (C) Transthyretin (TTR) staining, a marker for the choroid plexus, is absent, confirming the lack of these cell types in the spheres. Scale bar = 200 µM. HCPEpiC: human choroid plexus epithelial cells, NSF: neurospheres, DAPI: 4′,6-diamidino-2-phenylindole. PECAM1: Platelet endothelial cell adhesion molecule.

**Supplementary Figure 6: Complementary Inflammatory Response to the CSF in the SVZ-on-a-Chip Model**. This figure presents the qPCR analysis of cytokines that did not show statistically significant changes in expression levels following RBCL/OxyHb exposure in the SVZ-on-a-chip model. IL6 (Interleukin 6), CCL20 (C-C Motif Chemokine Ligand 20), PTGS2 (Prostaglandin-Endoperoxide Synthase 2); IL8: Interleukin 8, CCL2: C-C Motif Chemokine Ligand 2, ICAM1: Intercellular Adhesion Molecule 1, NF-κβ: Nuclear Factor Kappa B, NOS3: Nitric Oxide Synthase 3 (Endothelial NOS).

**Supplementary Figure 7. Raw microscopic fields used to assemble Figure 7A.**
