# Supplementary figures and images for: Subventricular Zone‐on‐a‐Chip: A Model to Study Neurogenesis Disruption in Neonatal Intraventricular Hemorrhage
Source: Adv Sci (Weinh). 2025 Oct 24;13(3):e02145. doi: 10.1002/advs.202502145 (PMC12806495; doi:10.1002/advs.202502145)

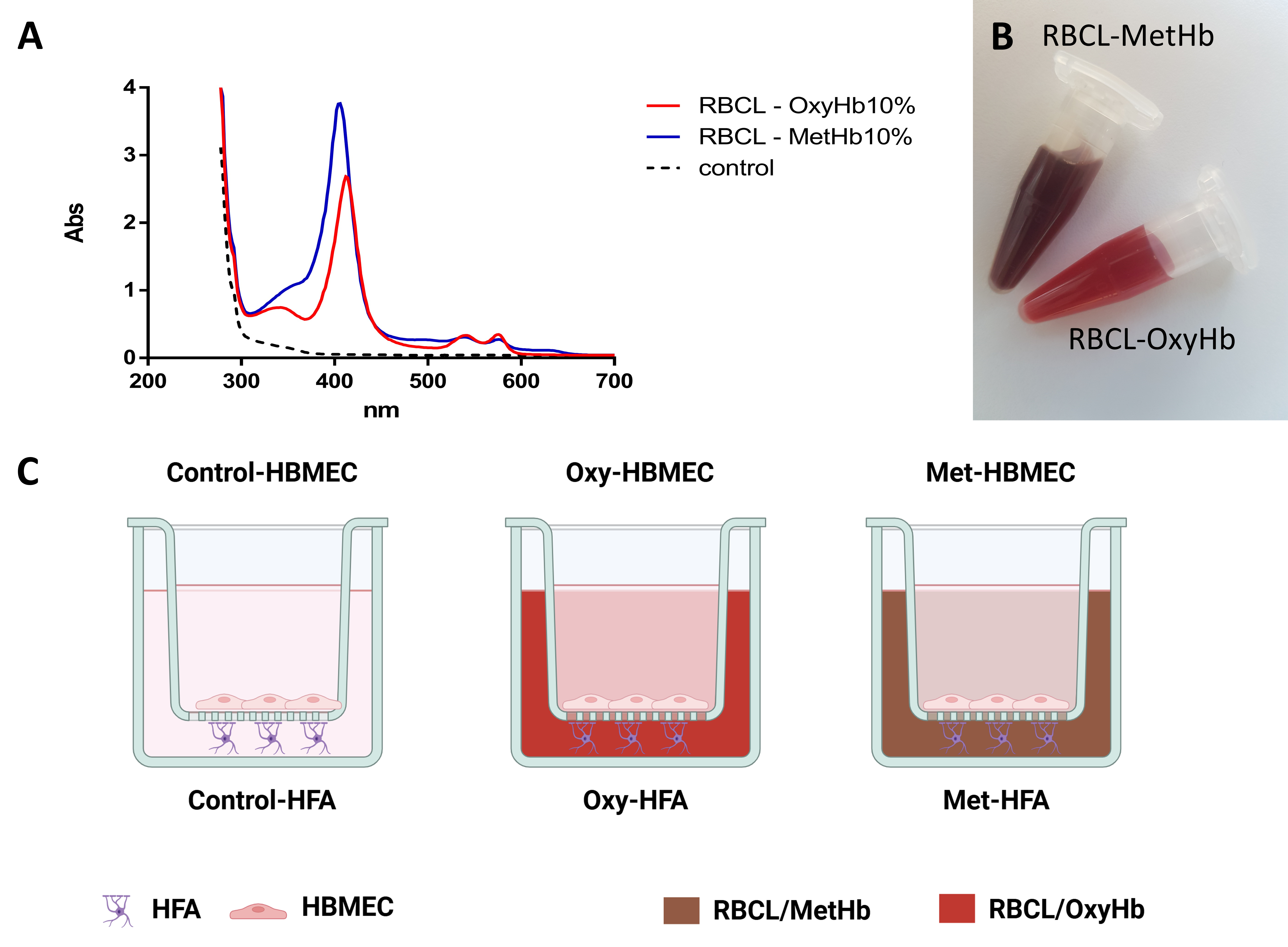

Supplement: Supplementary file 2 — Supplemental Figure 1‐7 [file ADVS-13-e02145-s003.zip › Supplementary Figure 1.tif]

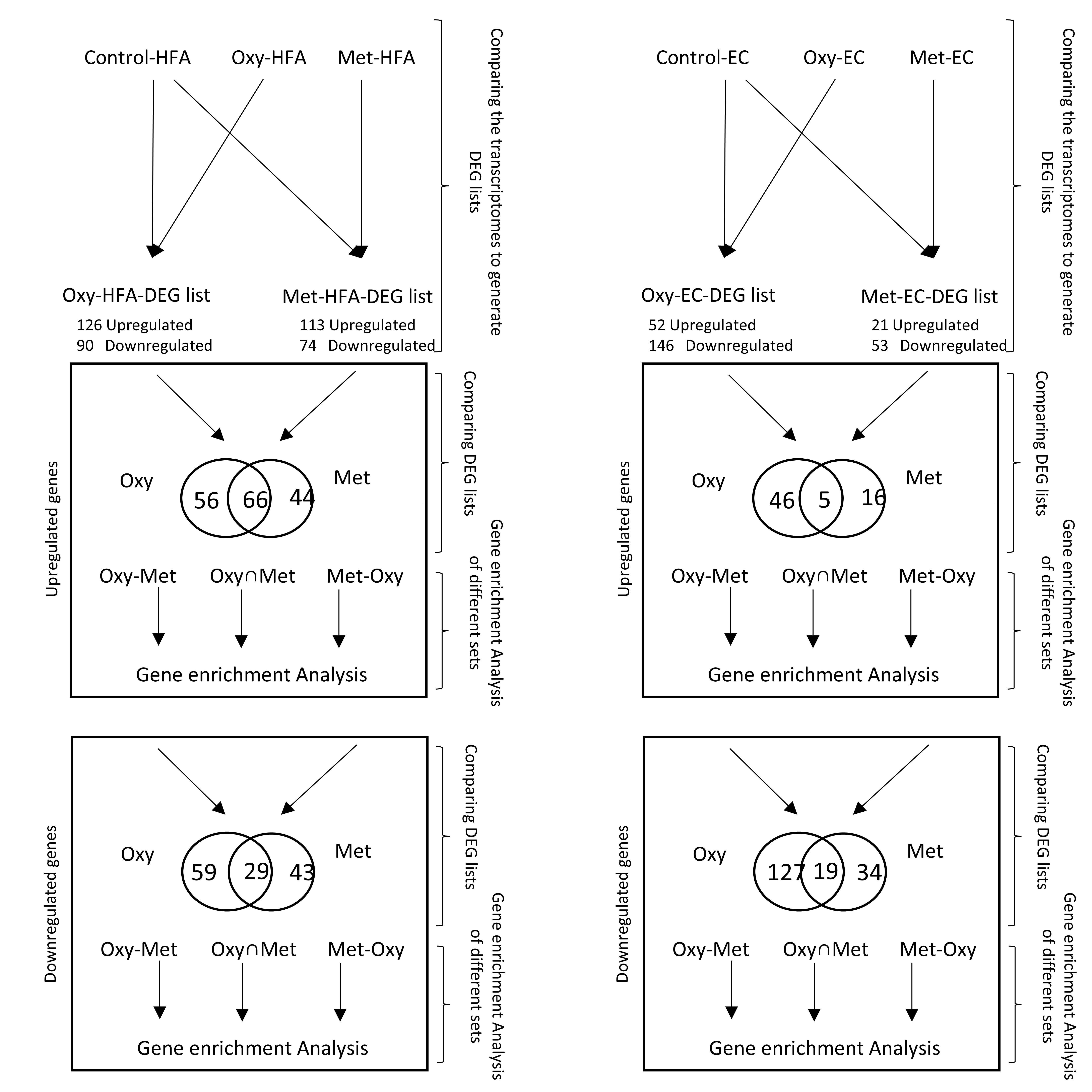

Supplement: Supplementary file 2 — Supplemental Figure 1‐7 [file ADVS-13-e02145-s003.zip › Supplementary Figure 2.tif]

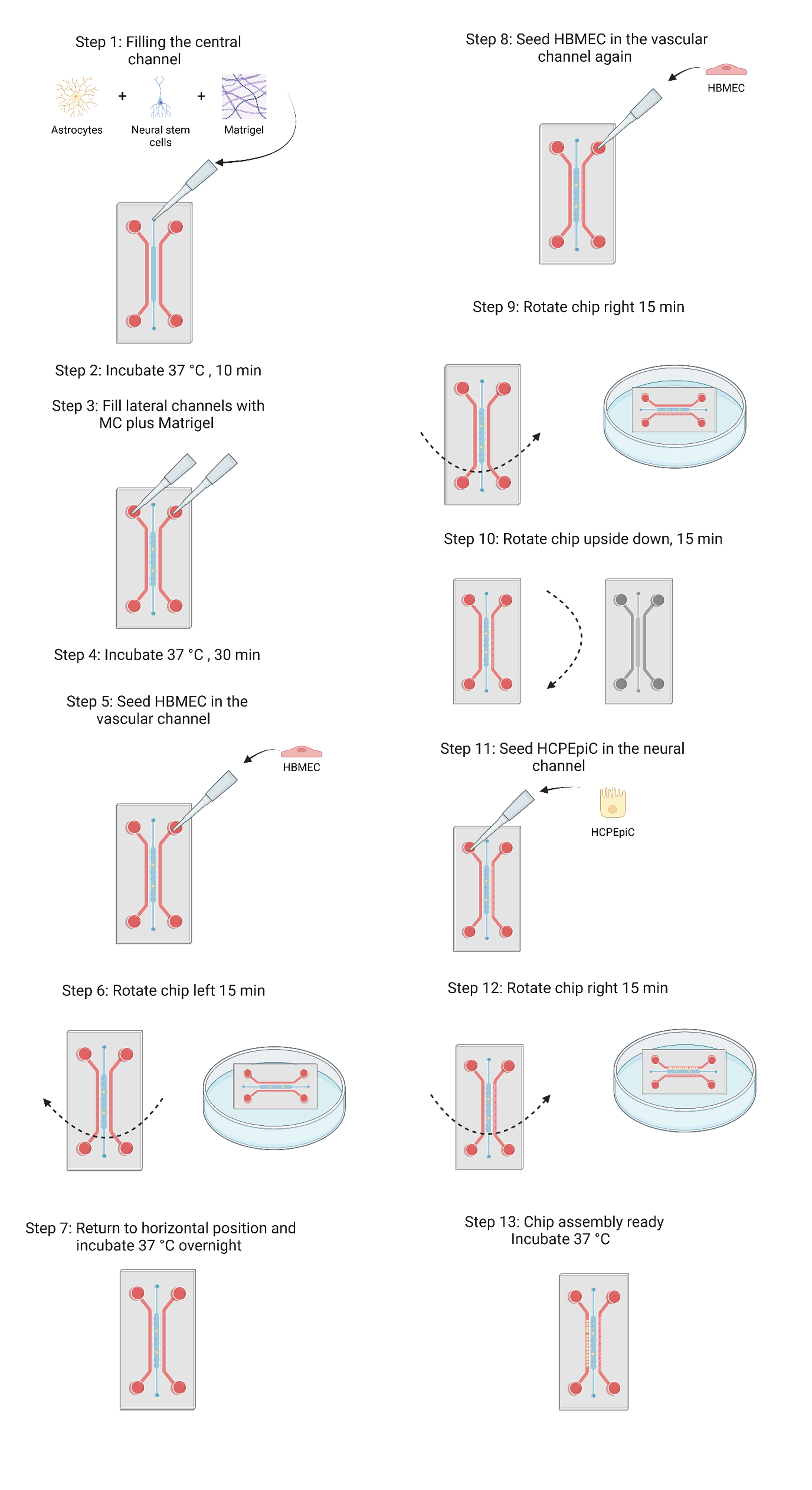

Supplement: Supplementary file 2 — Supplemental Figure 1‐7 [file ADVS-13-e02145-s003.zip › Supplementary Figure 3.tif]

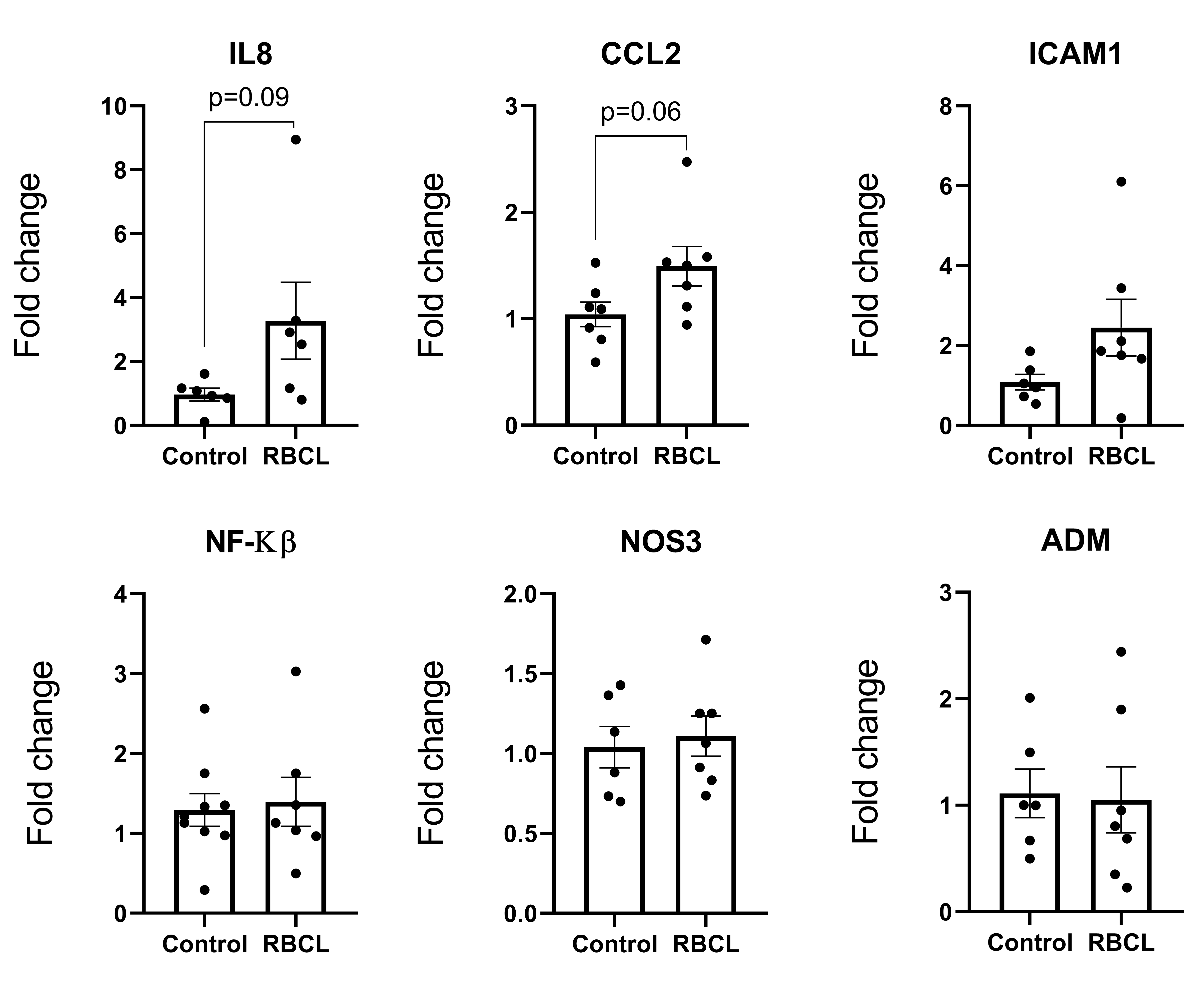

Supplement: Supplementary file 2 — Supplemental Figure 1‐7 [file ADVS-13-e02145-s003.zip › Supplementary Figure 4.tif]

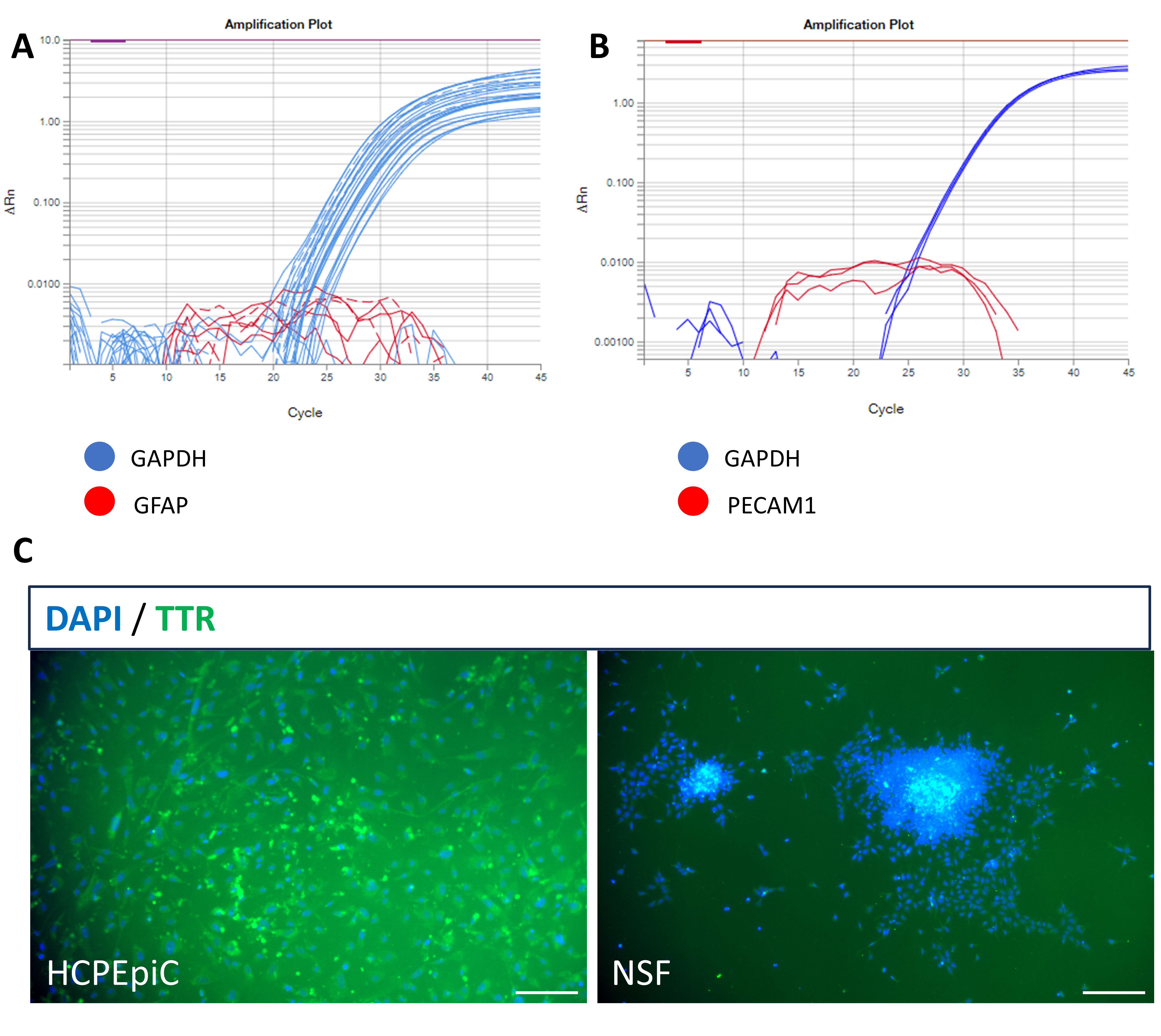

Supplement: Supplementary file 2 — Supplemental Figure 1‐7 [file ADVS-13-e02145-s003.zip › Supplementary Figure 5.tif]

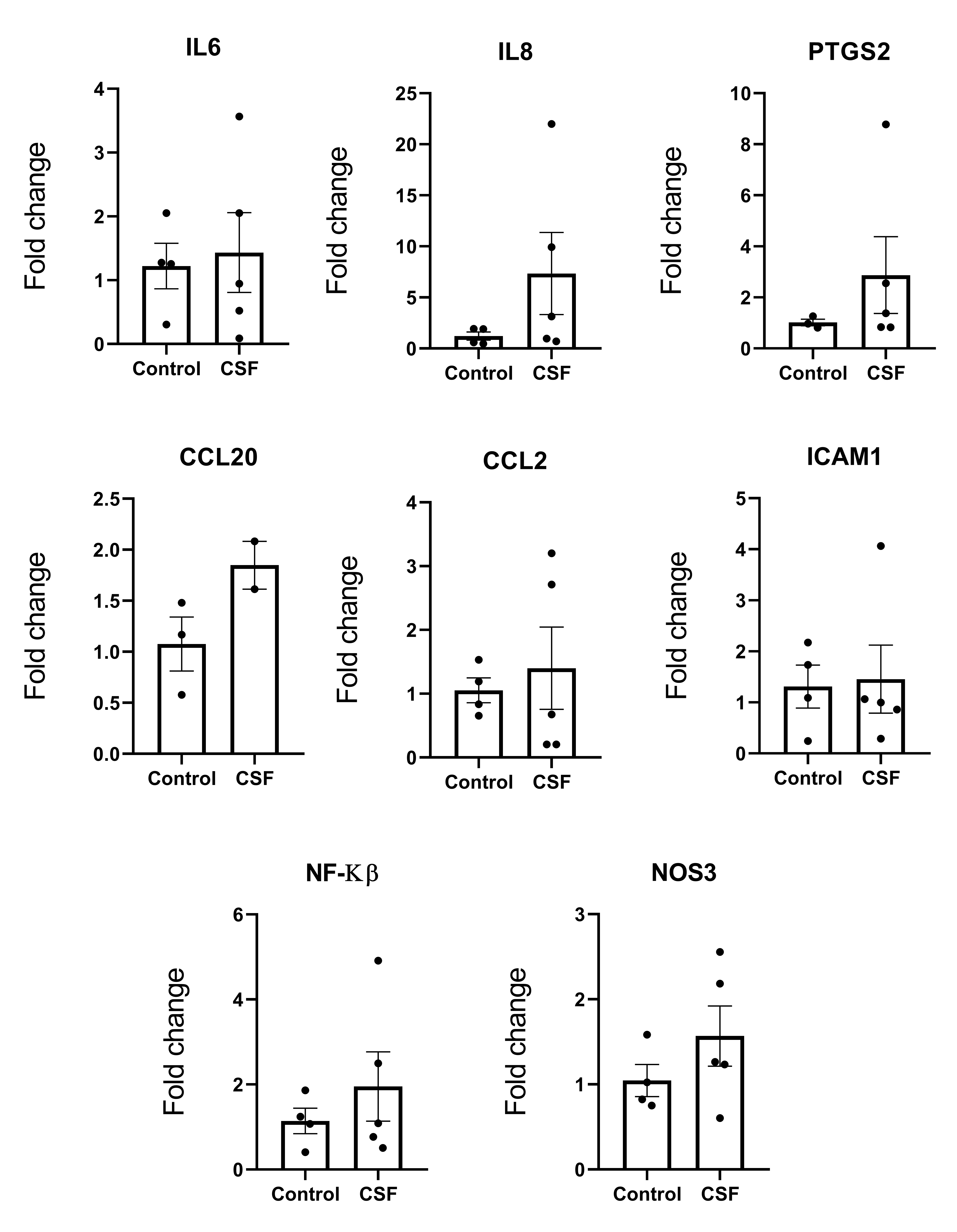

Supplement: Supplementary file 2 — Supplemental Figure 1‐7 [file ADVS-13-e02145-s003.zip › Supplementary Figure 6.tif]
