# Supplementary material for: Subventricular Zone‐on‐a‐Chip: A Model to Study Neurogenesis Disruption in Neonatal Intraventricular Hemorrhage
Source: Adv Sci (Weinh). 2025 Oct 24;13(3):e02145. doi: 10.1002/advs.202502145 (PMC12806495; doi:10.1002/advs.202502145)
Supplement: Supplementary file 4 — Supplemental Table 1 [file ADVS-13-e02145-s005.docx]

Supplementary Table 1. TaqMan probes

| **Gene** | **TaqMan Assay ID** |
| --- | --- |
| ACTB | Hs01060665_g1 |
| ADM | Hs00969450_g1 |
| CCL2 | Hs00234140_m1 |
| CCL20 | Hs00355476_m1 |
| DCX | Hs00167057_m1 |
| GFAP | Hs00909233_m1 |
| GAPDH | Hs02786624_g1 |
| HES1 | Hs00172878_m1 |
| HES3 | Hs01367669_g1 |
| HES5 | Hs01387463_g1 |
| HMOX1 | Hs01110250_m1 |
| ICAM1 | Hs00164932_m1 |
| IL1B | Hs00174097_m1 |
| IL6 | Hs00174131_m1 |
| IL8 | Hs00174103_m1 |
| Nanog | Hs02387400_m1 |
| Nestin | Hs04187831_g1 |
| NF1A | Hs00325656_m1 |
| NFKB | Hs00765730_m1 |
| NOS3 | Hs06610468_s1 |
| PECAM1 | Hs00169777_m1 |
| PTGS2 | Hs00153133_m1 |
| S100b | Hs00902901_m1 |
| SOX2 | Hs01053049_s1 |
| SOX9 | Hs00165814_m1 |
| TUBB3 | Hs00964962_g1 |
| VCAM1 | Hs01003372_m1 |
| NFE2L2 | Hs00975961_g1 |
| SOD2 | Hs00167309_m1 |
| GPX1 | Hs00829989_gH |
| CAT | Hs00156308_m1 |

ACTB : Actin Beta, ADM : Adrenomedullin, CCL2 : C-C Motif Chemokine Ligand 2, CCL20 : C-C Motif Chemokine Ligand 20, DCX : Doublecortin, GAPDH : Glyceraldehyde-3-Phosphate Dehydrogenase, HES1 : Hes Family BHLH Transcription Factor 1, HES3 : Hes Family BHLH Transcription Factor 3, HES5 : Hes Family BHLH Transcription Factor 5, HMOX1 : Heme Oxygenase 1, ICAM1 : Intercellular Adhesion Molecule 1, IL1B : Interleukin 1 Beta, IL6 : Interleukin 6, IL8 : Interleukin 8, Nanog : Nanog Homeobox, Nestin : Nestin, NF1A : Nuclear Factor I A, NFKB : Nuclear Factor Kappa B, NOS3 : Nitric Oxide Synthase 3, PTGS2 : Prostaglandin-Endoperoxide Synthase 2, S100b : S100 Calcium Binding Protein B, SOX2 : SRY-Box Transcription Factor 2, SOX9 : SRY-Box Transcription Factor 9, TUBB3 : Tubulin Beta 3 Class III, VCAM1 : Vascular Cell Adhesion Molecule 1¸ NFE2L2: Nuclear Factor, Erythroid Derived 2, Like 2 SOD2: superoxide dismutase 2, GPX1: glutathione peroxidase 1, CAT: catalase.
