# Supplementary material for: Subventricular Zone‐on‐a‐Chip: A Model to Study Neurogenesis Disruption in Neonatal Intraventricular Hemorrhage
Source: Adv Sci (Weinh). 2025 Oct 24;13(3):e02145. doi: 10.1002/advs.202502145 (PMC12806495; doi:10.1002/advs.202502145)
Supplement: Supplementary file 5 — Supplemental Table 2 [file ADVS-13-e02145-s006.docx]

Supplementary Table 2. Antibody Catalog with Suppliers and Fluorophore Labels

| **Antibody** | **Supplier** | **Catalog Number** |
| --- | --- | --- |
| DXC | Cell Signalling | 4604S |
| TUBB3 | Abcam | Ab522623 |
| S100b | Sigma | S2532 |
| Nestin | R&D | MAB1259 |
| VECAD | Cell Signalling | D87F2 |
| TTR (prealbumin) | Abcam | ab75815 |
| DAPI | Invitrogen | D1306 |
| Phalloidin 594 | Invitrogen | A12381 |
| Goat anti mouse 488 | Sigma | SAB4 600 238 |
| Goat anti rabbit 594 | Sigma | SAB4 600 107 |
| Goat anti rabbit 488 | Sigma | SAB4 600 044 |
| Goat anti mouse 633 | Sigma | SAB4 600 336 |

DXC: Doublecortin, TUBB3: Tubulin Beta 3 Class III, S100b: S100 Calcium Binding Protein B, VECAD: Vascular endothelial cadherin, TTR: transthyretin**,** DAPI: 4′,6-Diamidino-2-Phenylindole.
