# Supplementary material for: Subventricular Zone‐on‐a‐Chip: A Model to Study Neurogenesis Disruption in Neonatal Intraventricular Hemorrhage
Source: Adv Sci (Weinh). 2025 Oct 24;13(3):e02145. doi: 10.1002/advs.202502145 (PMC12806495; doi:10.1002/advs.202502145)
Supplement: Supplementary file 6 — Supplemental DataFile [file ADVS-13-e02145-s002.zip › Supplementary File 7..docx]

>TC0700009689.hg.1 [organism=Homo_sapiens] NOS3 5'-3'

CAGGATGGGCAGGATGGAGGGAGAAGGAAGGGACAGAGAGAAGGTCAGACAGAGGCAAGGGCTGAAGCTGAGGCCAGCACAGAAGCCACAGGAAGCCAGAGGCCAGACAGCCTGGGGCGGTGCCTGCACCGCAGAACTGGTCCCGGGCCGGGCAAGCAAGCACAGGGAGAGGTGGATCCCTGGGGGCTGTGGCTTTTTAAGCCTGGGCTTCCTCAGGGGCAGTGCTGCCTGTCTGGGGATCATGTCTGCAGTTGACAAGGGCTCGGTCTCCCCAGTGCCACACTGTTCAGGGCAGTGCTGCTGTCCCGGGGCCCAGGCTGGAGCTCAGCAGATTTGCCTTGATTGGAGGAGGAGGGCATCCTAGGAGGAGAGGGAGTGGGGGCTACCTCAGGGACGGGGAGGTCAGGCTGCAGAAACACATAGGCCCTGATTGGGAAGAAGGGAACGGAAAATAAGACTTAAAGAATTTAAACAAAAAGAGCCATTGCAGCGGGATGAGACCACATCATCAGGTTTTGGGAATAGGACTTTAGAGGCGTAGGATCCATTACAGCATCACCGAACCAGAAGCAGGAAGGCTGAGCTAAGCAGAGCAGCAGCAGTGGAGATAGGAAGGAAGGGAGGGAGGGGCCGAGGAAGGAAGGAAGAGATATAAGACTTCACACGCACCACAAAAGAAAGATTAACGGGACTTGGTGATATGAGGCTCAGCCAATCACGGGTGAGCCCTGCATTTCAAGCCTGGGACTGGCCCAGCAGTTTTCCAGCTGTGTGCCTGACCAGGAGTAGACGGGATCCACACCCTCCCAGGGATCTGCCCCGTGGGGTCCCCTCTGCCGCCCGAATTGTGCGTCCCTTCCCAGGAGCACTTACTATCTGCACGCACTTTGTGGAAAGCTAAGGGCTTTACATAAAGTATCTCATTTAATCTTCACCAGAACACAATGAGGTGTAAAGATGGGGAAACTGAGGCATGTCACTGTAAGTACGGGATTCGGAATTTGAATGCAGGTCTGAACACACAGACGCCTTCACAGAGCTACCGTGTGCCAAGCACTATGCTTCTCGGATCACGGGATTAACACGCACCAGATAAGGAACGATGCACCAATCAGGACGTGCAGAGAAAGAGCCAGCCGGGTCCCTGGGCCCAGCGGCCAATCCATGAAATGGGCTGGCGGAAAAGGTGCTGTCCTTGGCGCCGGCCTCAGCCACTGGGGCTGCCAACCCCCCAGGAGCAAGAC
